# Assessment of Genetic Diversity in *Secale cereale* Based on SSR Markers

**DOI:** 10.1007/s11105-015-0896-4

**Published:** 2015-06-06

**Authors:** M. Targońska, H. Bolibok-Brągoszewska, M. Rakoczy-Trojanowska

**Affiliations:** Department of Plant Genetics, Breeding, and Biotechnology, Warsaw University of Life Sciences, Warsaw, Poland

**Keywords:** Rye, *Secale cereale*, Genetic diversity, SSR markers

## Abstract

The primary aim of this study was to estimate genetic diversity among *Secale cereale* L. accessions using 22 previously published simple sequence repeat (SSR) markers. The plant material included 367 rye accessions comprising historical and contemporary cultivars, cultivated materials, landraces, and breeding strains from the Polish breeding company Danko. The studied accessions represented a wide geographical diversity. Several methods were employed to analyze genetic diversity among the *Secale cereale* L. accessions and to determine population structure: principal coordinate analysis (PCoA), neighbor-joining (NJ), and Bayesian clustering. We also defined a core collection of 25 rye accessions representing over 93 % of SSR alleles. The results of these analyses showed that accessions from the rye gene bank are clearly divergent in comparison with materials received directly from European breeding companies. Our findings suggest also that the genetic pool of current rye cultivars is becoming narrower during breeding processes. The selected panel of SSR markers performed well in detection of genetic diversity patterns and can be recommended for future germplasm characterization studies in rye.

**Electronic supplementary material:**

The online version of this article (doi:10.1007/s11105-015-0896-4) contains supplementary material, which is available to authorized users.

## Introduction

The genus *Secale* comprises typical representatives of the Mediterranean flora, and its members are widely distributed from Central Europe and the Western Mediterranean through the Balkans, Anatolia, Israel, and the Caucasus to Central Asia. An isolated population also occurs in South Africa. The genus includes annuals, short-lived and long-lived perennials, wild, weedy, and cultivated taxa (Sencer and Hawkes 1980).

Common rye (*Secale cereale* L.) is one of the most important cereal crops cultivated in Eastern and Northern Europe. It is characterized by the ability to produce high yields even when grown under environmental stress conditions, i.e., low temperatures, drought, and low soil fertility. The presence of disease resistance genes reduces the need for intensive chemical protection of this crop (Schlegel and Melz 1996; Korzun et al. 2001). Moreover, rye offers high contents of many nutritionally favorable compounds such as a whole suite of minerals (Zn, Fe, P), beta-glucans, resistant starch, and bioactive compounds. Rye products are characterized by a high level of dietary fiber (Andersson et al. 2009) that may contribute to positive health effects (Rosén et al. 2011).

Relatively good progress in rye breeding has been achieved in the case of hybrid cultivars (Geiger and Miedaner 1999). The two heterotic pools Carsten and Petkus were selected as the most promising heterotic pattern. During hybrid breeding, parental inbred lines were developed by recurrent selfing of plants from both pools, or inbred lines were generated by second-cycle breeding (Fischer et al 2010). These processes contributed to the reduction of genetic diversity within the heterotic pools. The consequences of such narrowing of germplasm diversity in breeding programs are a decrease in selection gain and an increase in susceptibility to biotic and abiotic stresses, coupled with the threat of further genetic erosion (Smith et al. 2004). To prevent genetic erosion, it is important to continuously broaden the genetic base of the established heterotic pools. However, a major difficulty preventing the exploitation of genetic resources in hybrid rye breeding is the prevalence of self-incompatibility. Furthermore, in the absence of any prior knowledge about the relationships within heterotic pools, testcrosses with both such pools have to be developed and evaluated (Fischer et al. 2010). The implementation of modern methods based on knowledge of the genetic diversity within the species *S. cereale* could significantly improve the efficiency of breeding, and thus increase the competitiveness of rye.

Studies on relationships within (Matos et al. 2001; Bolibok et al. 2005; Burger et al. 2006) and between *Secale* species (Shang et al. 2006; Skuza et al. 2007) have been carried out using a variety of different methods. The molecular tools employed in these analyses include PCR-RFLP (Isik et al. 2007), RAPD (Matos et al. 2001; Bolibok et al. 2005), ISSR (Bolibok et al. 2005), AFLP (Chikmawati et al. 2005 and 2012), SAMPL (Bolibok et al. 2005), SSR (Bolibok et al. 2005; Shang et al. 2006; Fu et al. 2010), DArT (Bolibok-Brągoszewska et al. 2009, 2014), and isoenzymatic markers (Matos et al. 2001; Burger et al. 2006). However, in the most cases, the number of accessions included in these analyses has been relatively low. One exception is our recent study (Bolibok-Brągoszewska et al. 2014) where 379 rye accessions, including cultivars, landraces, and wild relatives, were used. The reported results showed that cultivated rye forms, especially modern cultivars, are genetically similar, while relatively high genetic diversity was observed between landraces. Moreover, the lack of correlation between clustering of improved accessions and geographic origin was observed. These results may suggest that rye accessions from diverse geographic regions have common genetic background and indicate the extensive germplasm exchange. The DArT method employed by Bolibok-Brągoszewska et al. (2014) is widely used in studies on genetic diversity in crops. It permits the simultaneous detection of several thousand DNA polymorphisms throughout the whole genome. However, in the case of rye, which is heterogenic and characterized by a high level of heterozygosity, some information about genetic diversity may be lost during genotyping using dominant DArT markers. Simple sequence repeat (SSR) markers, which are amplified microsatellite sequences, are very useful in genetic studies due to their multi-allelic nature, codominant inheritance, and high informativeness (Rakoczy-Trojanowska and Bolibok 2004). Rye SSR markers were first developed over 10 years ago (Saal and Wricke 1999; Hackauf and Wehling 2002) and have also been used in studies on genetic diversity (Shang et al. 2006; Bolibok et al. 2005). Recently, a considerable number of rye SSR markers was developed by Haseneyer et al. (2011), but their utility in genetic diversity studies has yet to be examined.

In the literature, there are several examples of the usefulness of SSRs in a comparison with other marker systems like DArT and SNP (Laidò et al. 2013; Hurtado et al. 2008; Simko et al. 2012). Such analyses were conducted in tetraploid wheat (Laidò et al. 2013), cassava (Hurtado et al. 2008), and sugar beet (Simko et al. 2012). In genetic studies of the genus *Secale*, SSR data would be useful to confirm results obtained using DArT markers (Bolibok-Brągoszewska et al. 2014) and extend our knowledge about genetic diversity structure.

The main purpose of the present study was to evaluate genetic diversity within a broad population consisting of *S. cereale* L. accessions using SSR markers. We also wanted to broaden the results of our earlier genetic diversity analysis obtained using the DArT system.

## Materials and Methods

### Plant Material

The plant material consisted of 367 *S. cereale* ssp*. cereale* accessions from different parts of the world: 90 population cultivars, 46 cultivated materials, and 155 landraces obtained from the PAS BG in Powsin (Poland); 27 population cultivars, 12 hybrid cultivars, and 26 breeding strains from European breeding companies; and 11 rye accessions from the collection of A. Lukaszewski from the University of California, Riverside (USA). Out of 367 accessions used in this study, 362 were examined previously using DArT markers (Bolibok-Brągoszewska et al. 2014). The assembled plant material was selected to represent genetic and geographical variation of cultivated rye. Polish rye inbred line L318 and wheat cultivar Chinese Spring were used as references. Detailed information about rye accessions used including source of seeds, improvement status, and region of origin can be found in Online Resource 1.

### Plant Propagation and DNA Isolation

Each rye accession used in genetic diversity analysis was represented by 96 individual plants. Plants were grown in multi-trays in an air-conditioned glasshouse. Two-week-old leaves were collected and lyophilized. Total DNA was isolated using a Mag-Bind Plant DNA 96 kit (Omega Bio-Tek, Norcross, USA). Equal amounts of DNA from the 96 plants representing each accession were pooled into one sample. DNA purity and concentration were evaluated using a NanoDrop 2000 spectrophotometer (Thermo Scientific, Waltham, USA).

### SSR Assays

Forty-two public SSR markers (Hackauf and Wehling 2002; Saal and Wricke 1999) (Online Resource 2) were included in the genetic diversity study. The initial selection of publicly available markers was made based on literature data. Markers representing all chromosomes and characterized by a high polymorphism level were selected (Bolibok et al. 2005; Shang et al. 2006; Hackauf et al. 2009).

The analysis of genetic diversity was performed in two steps. In the first step, SSR markers were used to analyze six highly divergent rye accessions, selected based on the results of DArT marker analysis (Bolibok-Brągoszewska et al. 2014): cultivars Dańkowskie Nowe and Zima from PAS BG, landrace M1-72-73-321 from PAS BG, cultivar Prima from the collection of A. Lukaszewski, cultivar Palazzo from KSW Lochow, and cultivar Riihi from the company Boreal. Based on the results of this initial screening, 22 SSR markers were selected for the second step of the analysis including all 367 rye accessions (Table 1). The marker selection criteria were the presence of good quality and polymorphic products. Moreover, only markers amplifying a single locus were selected, based on the results obtained using the inbred line L318.

**Table 1:** Parameters characterizing 22 SSR markers used in genotyping

SSR marker	Chromosome	PIC	No. of alleles	Proportion of polymorphic alleles	Frequent alleles (>50 %) [%]	Rare alleles (<5 %) [%]
SCM171	1R	0.44	3	0.67	66.66	0.00
SCM107	1R	0.62	5	1.00	40.00	60.00
SCM009	1R	0.43	4	0.50	75.00	0.00
SCM041	2R	0.76	12	1.00	33.33	33.33
SCM073	2R	0.43	8	1.00	75.00	12.50
SCM075	2R	0.59	6	0.83	50.00	16.60
SCM118	2R	0.56	5	1.00	60.00	40.00
SCM162	3R	0.37	5	1.00	100.00	0.00
SCM112	3R	0.28	4	1.00	100.00	0.00
SCM139	4R	0.75	14	1.00	35.71	21.43
SCM155	4R	0.60	5	0.60	40.00	20.00
SCM101	4R	0.88	9	1.00	22.22	0.00
SCM138	5R	0.64	4	1.00	50.00	0.00
SCM109	5R	0.73	6	1.00	50.00	33.33
SCM172	5R	0.68	5	1.00	40.00	20.00
SCM152	5R	0.93	9	1.00	11.11	55.55
SCM180	6R	0.54	6	1.00	66.66	0.00
SCM168	6R	0.43	6	1.00	83.33	0.00
SCM002	6R	0.46	11	0.82	63.63	27.27
SCM028	6R	0.77	8	1.00	37.50	50.00
SCM050	7R	0.18	2	1.00	100.00	0.00
SCM063	7R	0.46	5	1.00	60.00	40.00
Summary		Mean PIC	No. of alleles	Mean proportion of polymorphic alleles	Mean % of frequent alleles	Mean % of rare alleles
		0.57	142	0.93	57.28	19.55

All PCRs were conducted in 15 μl volumes containing 50 ng of genomic DNA, 0.5 U of DreamTaq polymerase (Thermo Scientific, Waltham, USA), 1.5x DreamTaq buffer, 0.2 mM dNTP’s, 0.5 mM MgCl_2_, and 0.2 μM of each primer. Amplification was carried out in a Veriti 96 Thermal Cycler (Applied Biosystems, Foster City, USA) under conditions specified by the high-stringency protocol of Pillen et al. (2000). The amplified products were separated on 6 % denaturing polyacrylamide gels and visualized by silver staining (Benbouza et al. 2006). The consistency of allele sizing across gels and accessions was ensured using 10 bp Ladder and 50 bp Ladder (Thermo Scientific, Waltham, USA). In case of amplification failure, analyses were repeated until a clear result was obtained.

### Data Analysis

For ease of analysis, we labeled rye accessions according to their geographical origin, source of the seeds, and assignation to one of the heterotic pools. We also conducted separate analyses only for accessions from breeding companies and from PAS BG.

Based on the PCR results, a binary matrix was constructed, where the presence of an amplified product was scored as 1 and the absence of a product as 0. Because the templates used in SSR analysis were pooled DNA from 96 plants and more than two alleles per sample were observed, separate bands were treated as individual alleles. The bands were scored as present or absent, and allele frequencies within individual accessions were not established. The number of alleles, number of polymorphic alleles, proportion of polymorphic products (the number of polymorphic alleles divided the total number of alleles), and percentage of frequent (appearing in >50 % of rye accessions) and rare (appeared in <5 % of accessions) alleles were counted. Polymorphic information content (PIC) was calculated according to the formula of Roy et al. (2002).

The total number of alleles, number of alleles with a frequency of <5 %, number of private alleles, number of alleles found in more than 25 and 50 % of the accessions from subgroups created based on source of seeds and improvement status, mean diversity, and unbiased diversity were calculated using GenAlEx 6.501 (Peakall and Smouse 2012).

Based on the binary matrices, genetic similarity (GS) was calculated using Jaccard and Dice coefficients with NTSYSpc 2.1 software (Rohlf 2000). Correlations between two similarity matrices were demonstrated by Mantel’s test applied in NTSYSpc.

STRUCTURE software (Pritchard et al. 2000) was used to infer population structure. To identify the number of populations (*K*) capturing the major structure in the data, a burn-in period of 100,000 Markov Chain Monte Carlo (MCMC) iterations was used, with 100,000 run length and admixture model following the Hardy-Weinberg equilibrium. Five independent runs were performed for each simulated value of *K*, ranging from 1 to 20. The most likely number of *K* was determined using the DeltaK method in Structure Harvester (Earl and von Holdt 2011) with the Evanno correction (Evanno et al. 2005). Permutations of the output of STRUCTURE analysis were calculated with CLUMPP (Jakobsson and Rosenberg 2007) using independent runs to obtain a consensus matrix. Bar graphs of the population structure results were generated with Distruct (Rosenberg 2004).

A 1-GS distance matrix was used to construct a dendrogram in MEGA 5.2 (Tamura et al. 2011) using the neighbor-joining (NJ) clustering method. GS matrix was used to conduct principal coordinate analysis (PCoA) in NTSYSpc 2.1.

The significance of differences between populations indicated by STRUCTURE, between groups representing different geographical origins and between accessions groups from different sources and with a different improvement status were tested by analysis of molecular variance (AMOVA) in GenAlEx 6.501.

For core collection sampling, the maximization (*M*) algorithm implemented through a modified heuristic algorithm in PowerCore 1.0 was used (Kim et al. 2007).

## Results

### SSR Marker Informativeness

The first step in this analysis employing 42 publicly available SSRs resulted in establishment of the amplified product length (Online Resource 2) and quality.

For the second step of the analysis, 22 SSR markers were selected. In total, 142 bands were detected in 367 rye accessions, and 132 bands were polymorphic (93 %) (Table 1). The average PIC value for all markers used was 0.57. The highest PIC value (0.93) was obtained for SCM152, and the lowest PIC (0.18) was determined for SCM050. In the case of the markers with the highest PIC value, frequent alleles appeared in ≤40 % of accessions.

Analysis of the total allele pattern within groups of accessions sorted according to the source of the seeds and improvement status (Table 2) showed that the highest number of detected bands (130) was observed for landraces (PAS BG) and the lowest for breeding strains from Danko (93). The number of bands with a frequency of <5 % was the highest for landraces (18) and the lowest for the collection of A. Lukaszewki (0). There were no unique bands in the group of breeding strains from Danko, while the highest numbers of unique bands (4) within groups were observed for the collection of A. Lukaszewski and landraces. Mean heterozygosity was also the lowest for breeding strains from Danko (*h* = 0.113), while this value was nearly two fold higher for the collection of A. Lukaszewski (*h* = 0.218).

**Table 2:** Total allele pattern within groups of rye accessions according to the source of seeds

	Cultivars BG PAS	Cultivated materials BG PAS	Landraces BG PAS	Collection of A. Lukaszewski	Cultivars from breeding companies	Breeding materials from Danko
No. accessions	90	46	155	11	39	26
No. alleles	120	124	130	109	113	93
No. rare alleles <5 %	17	15	18	0	13	4
No. private alleles	1	1	4	4	2	0
No. frequent alleles (>25 %)	120	124	130	109	113	93
No. frequent alleles (>50 %)	105	108	112	101	102	92
Mean *h* (SE)	0.167 (0.015)	0.159 (0.014)	0.193 (0.015)	0.218 (0.016)	0.170 (0.014)	0.113 (0.013)
Mean uh (SE)	0.169 (0.015)	0.163 (0.015)	0.194 (0.015)	0.240 (0.017)	0.174 (0.015)	0.118 (0.014)
Mean GS	0.717	0.743	0.678	0.630	0.693	0.803
GS range	0.459–0.888	0.575–0.892	0.441–0.929	0.439–0.784	0.255–0.918	0.610–0.932

No. alleles = no. of different bands

No. rare alleles (<5 %) = no. of different bands with a frequency <5 %

No. private alleles = no. of bands unique to a given group

No. frequent alleles (>25 %) = no. of alleles found in 25 % or more accessions in the group

No. frequent alleles (>50 %) = no. of alleles found in 50 % or more accessions in the group

*h* = diversity = 1 − (*p*^2 + *q*^2)

uh = unbiased diversity = (*N*/(*N −* 1)) × *h*

Where for haploid binary data, *p* = allele freq. and *q* = 1 − *p*

*SE* statistic error

### Genetic Similarity Analysis

Mantel’s test showed a strong positive correlation between Jaccard and Dice coefficient matrices with *r* = 0.991 and *P* = 0.001 (Online Resource 3), so only the Jaccard coefficient matrix was used in subsequent analyses. The highest GS (0.932) was observed between breeding strains SZK44 and SZK85 from Danko, and breeding strain SZK44 from Danko, whereas the lowest GS (0.255) was observed between cultivar Gonello F1 (KWS Lochow) and cultivar Dańkowskie Złote (Danko). The average GS value was 0.664. The average GS for accessions labeled according to the source of seeds ranged from 0.8 for breeding strains from Danko to 0.63 for the collection of A. Lukaszewski. The table with GS values can be found in the Online Resource 4.

### Bayesian Model-Based Clustering

**Fig. 1:**
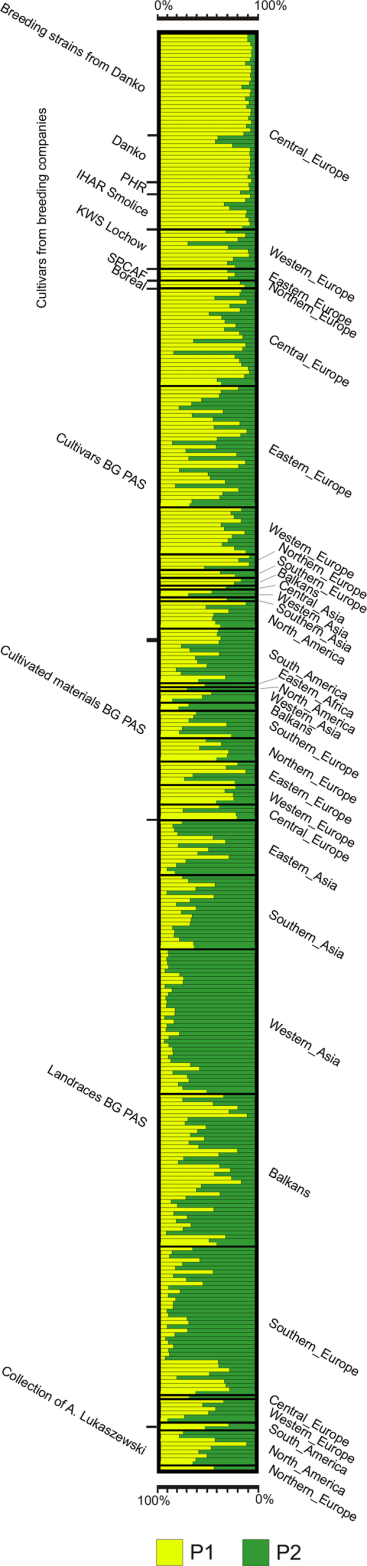
STRUCTURE plot showing the population structure of 367 rye accessions with *K* = 2 clusters, based on 22 SSR markers

After analysis of the population structure of all rye accessions using STRUCTURE with Evanno correction, the peak of Δ*K* was observed for *K* = 2 (Online Resource 5). This suggested the presence of two main model-based populations, which were visualized in the graph (Fig. 1). In total, 270 (74 %) rye accessions were assigned to one of the two model-based populations, with more than 65 % of their inferred ancestry derived from one of the respective populations. Populations one (P1) and two (P2) consisted of 144 (39 %) and 126 (34 %) accessions, respectively. The remaining 97 accessions (26 %) had mixed ancestry (P1P2).

Population P1 contained mainly accessions from breeding companies (43 %) and cultivars from PAS BG (34 %), especially from Western and Central Europe. Only cultivar Gonello (KWS Lochow) belonged to the P2 population. Breeding strains from the company Danko were the most homogeneous group and were characterized by an inferred ancestry level of >80 %. The P1 population also contained landraces, cultivated materials from European countries, and accession from the A. Lukaszewski collection.

Population P2 was comprised mainly of landraces from PAS BG (79 %), while the representation of cultivars and cultivated materials from PAS BG was relatively lower: 8 and 10 %, respectively. The geographical origins of the majority of accessions classified as P2 were Asia, the Balkans, and Southern Europe. The remaining P2 accessions were cultivars and cultivated materials originating from Eastern and Central Europe, and South and North America. Two accessions from the A. Lukaszewski collection originating from North America were also assigned to P2.

Accessions characterized by mixed ancestry were mainly from PAS BG (90 %). The geographical origin of accessions with mixed ancestry was very diverse.

### Cluster and Principal Coordinate Analyses

**Fig. 2:**
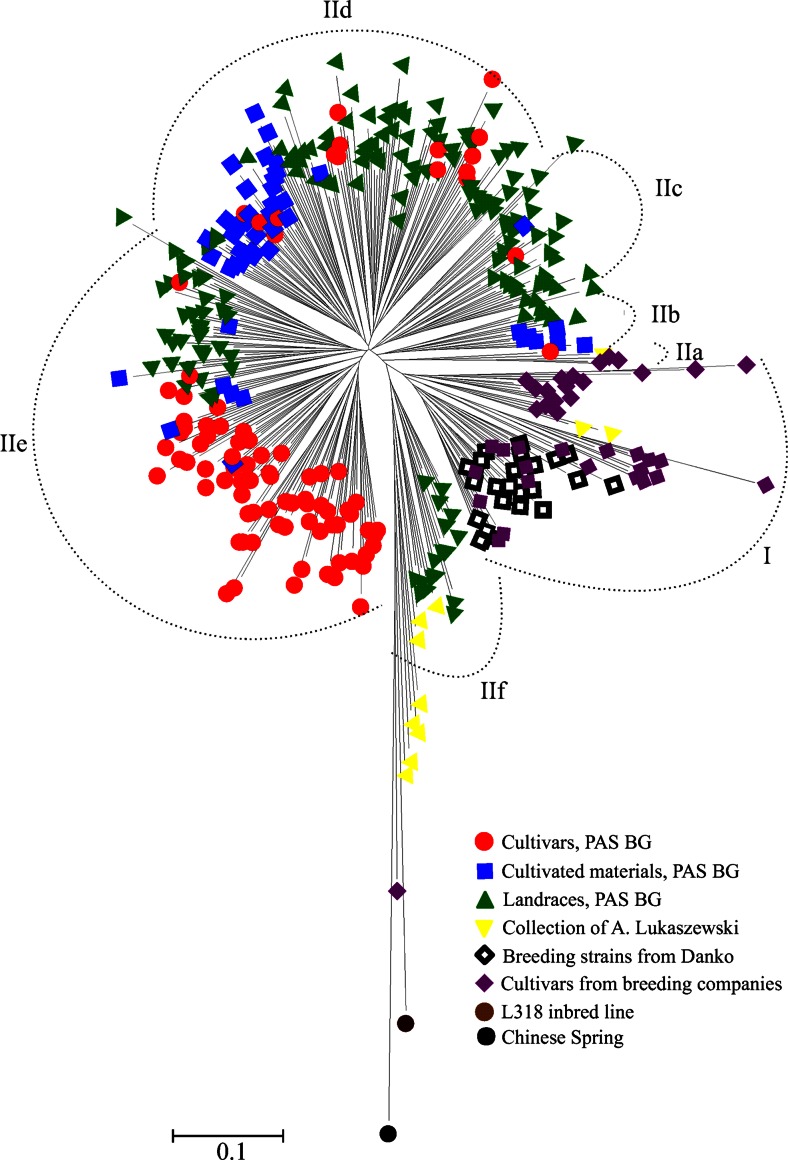
NJ dendrogram showing genetic relationships among 367 rye accessions. Accessions labeled according to the source of seeds

**Fig. 3:**
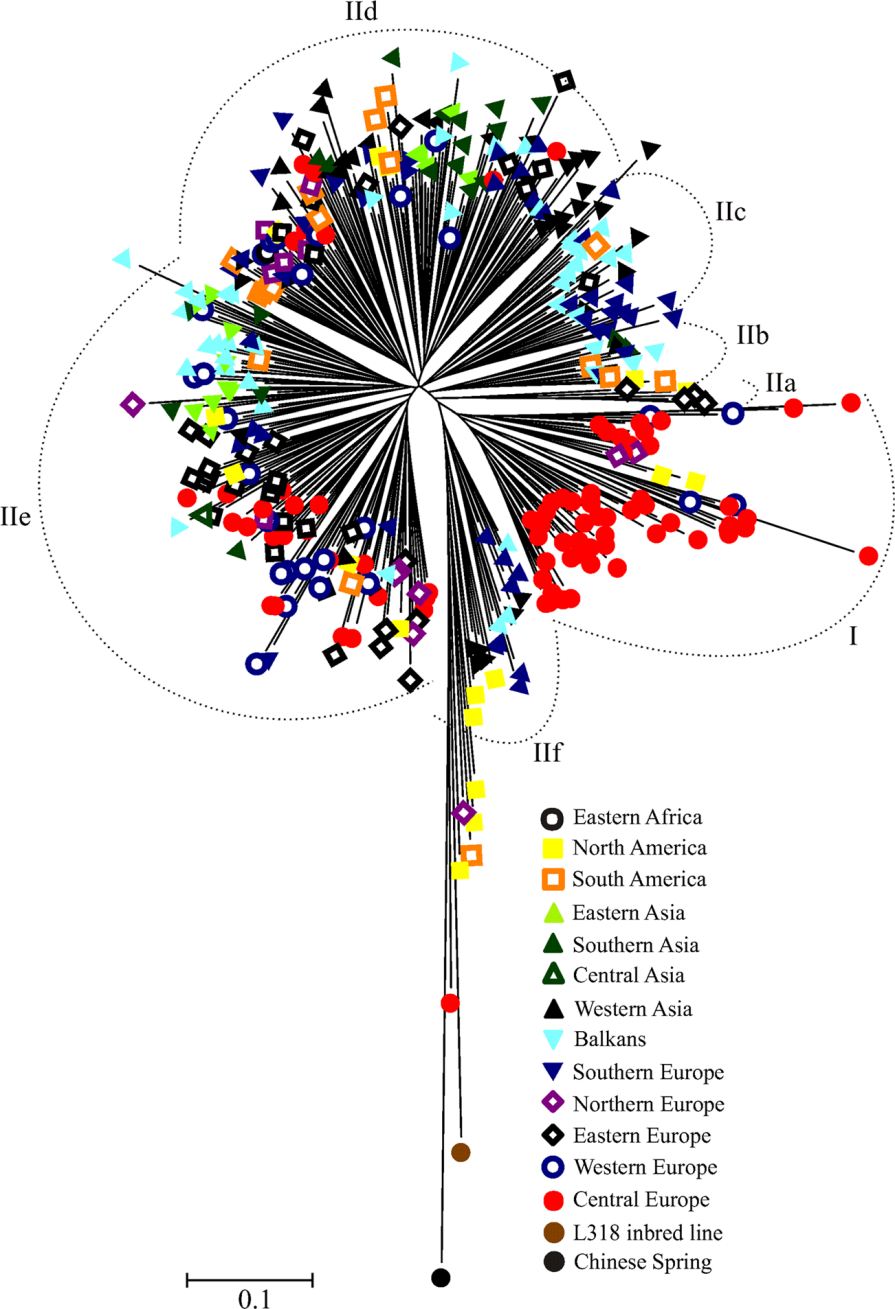
NJ dendrogram showing genetic relationships among 367 rye accessions. Accessions labeled according to the geographic origin of plants

Several clusters were visible in the NJ dendorgram (Figs. 2 and 3). To simplify the description of the results, we distinguished two major clusters: I and II, by separating the longest branch into two, and further divided cluster II into six subclusters: IIa–IIf. Concerning the source of seeds and improvement status (Fig. 2), cluster I was comprised of modern cultivars, breeding, strains and two accessions form A. Lukaszewski collection. Cluster II contained mainly accessions from PAS BG. The most dispersed group was landraces which occurred in five subclusters (IIb, IIc, IId, IIe, and IIf). The majority of cultivars from PAS BG were closely clustered in one subcluster (IIe). Cultivated materials from BG PAS were also grouped mainly into one subcluster (IId). The collection of A. Lukaszewski was located mostly in subcluster IIf; however, relatively longer tree branches indicated higher genetic diversity within these accessions. Labeling according to the geographical origin (Fig. 3) showed that the majority of Asian, South European, and in Balkan accessions were grouped in cluster II, although they were dispersed among different subclusters. Accessions from Central Europe were located mainly in cluster I. On the other hand, accessions from Western and Eastern Europe were clustered in subcluster IIe.

**Fig. 4:**
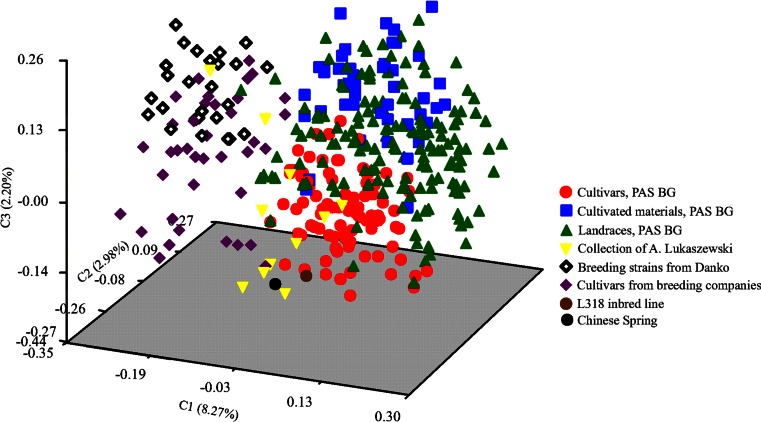
PCoA of 367 rye accessions based on 22 SSR markers. Accessions labeled according to the source of seeds

**Fig. 5:**
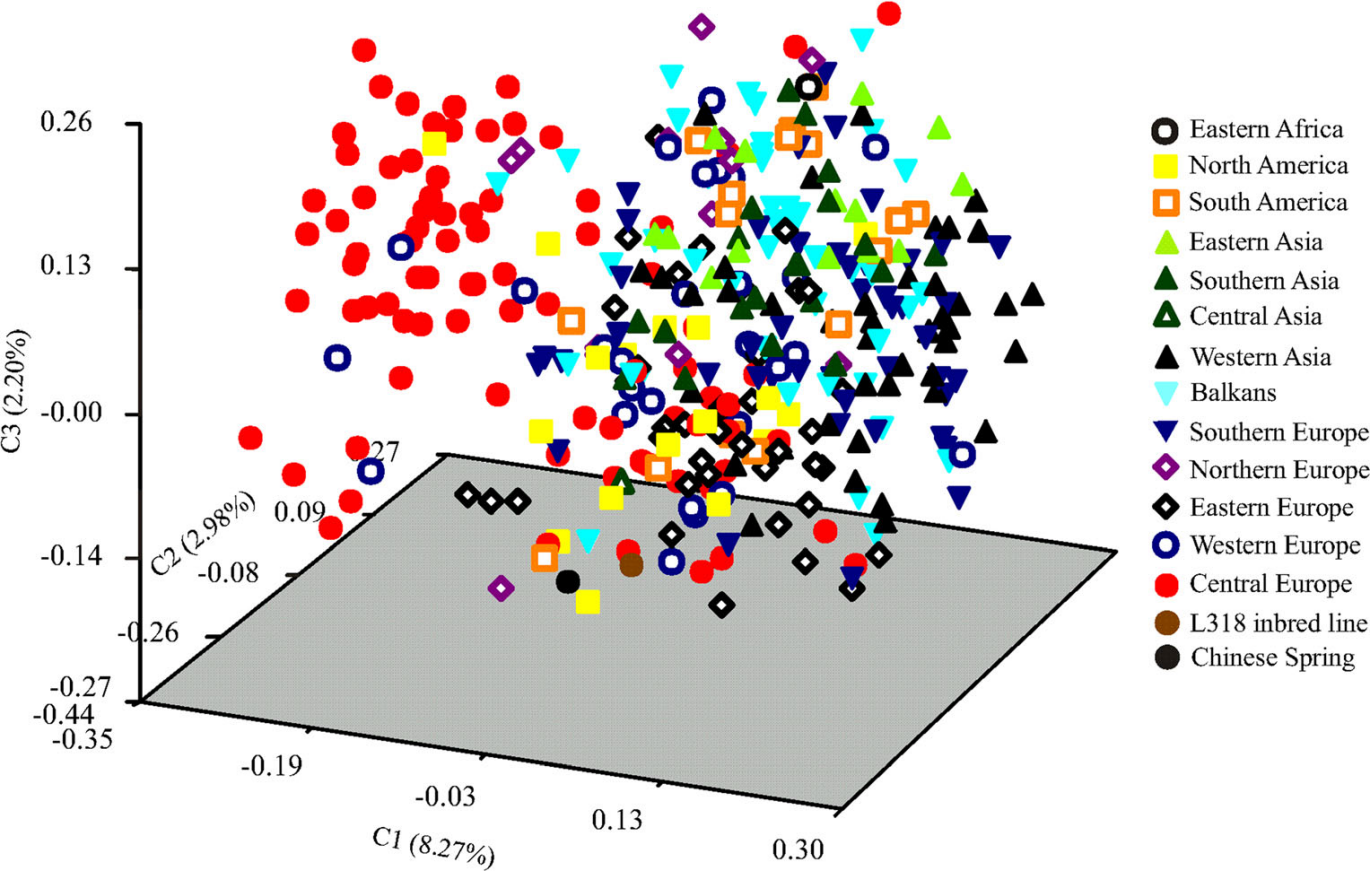
PCoA of 367 rye accessions based on 22 SSR markers. Accessions labeled according to the geographic origin of plants

Two groups could be distinguished in the PCoA plot (Figs. 4 and 5). Accessions from breeding companies were noticeably distinct from accessions from PAS BG (Fig. 4). The most dispersed and diverse group consisted of landraces, whereas cultivated materials and cultivars from PAS BG were more tightly grouped. The collection of A. Lukaszewski was the most distinct group, but two of its accessions were placed close to the cultivars from breeding companies. Labeling based on the geographical origin (Fig. 5) showed that one the two groups mostly represented rye accessions from Central Europe and the other comprised accessions representing different geographical origins. Accessions from Central and Western Europe were grouped close to each other.

**Fig. 6:**
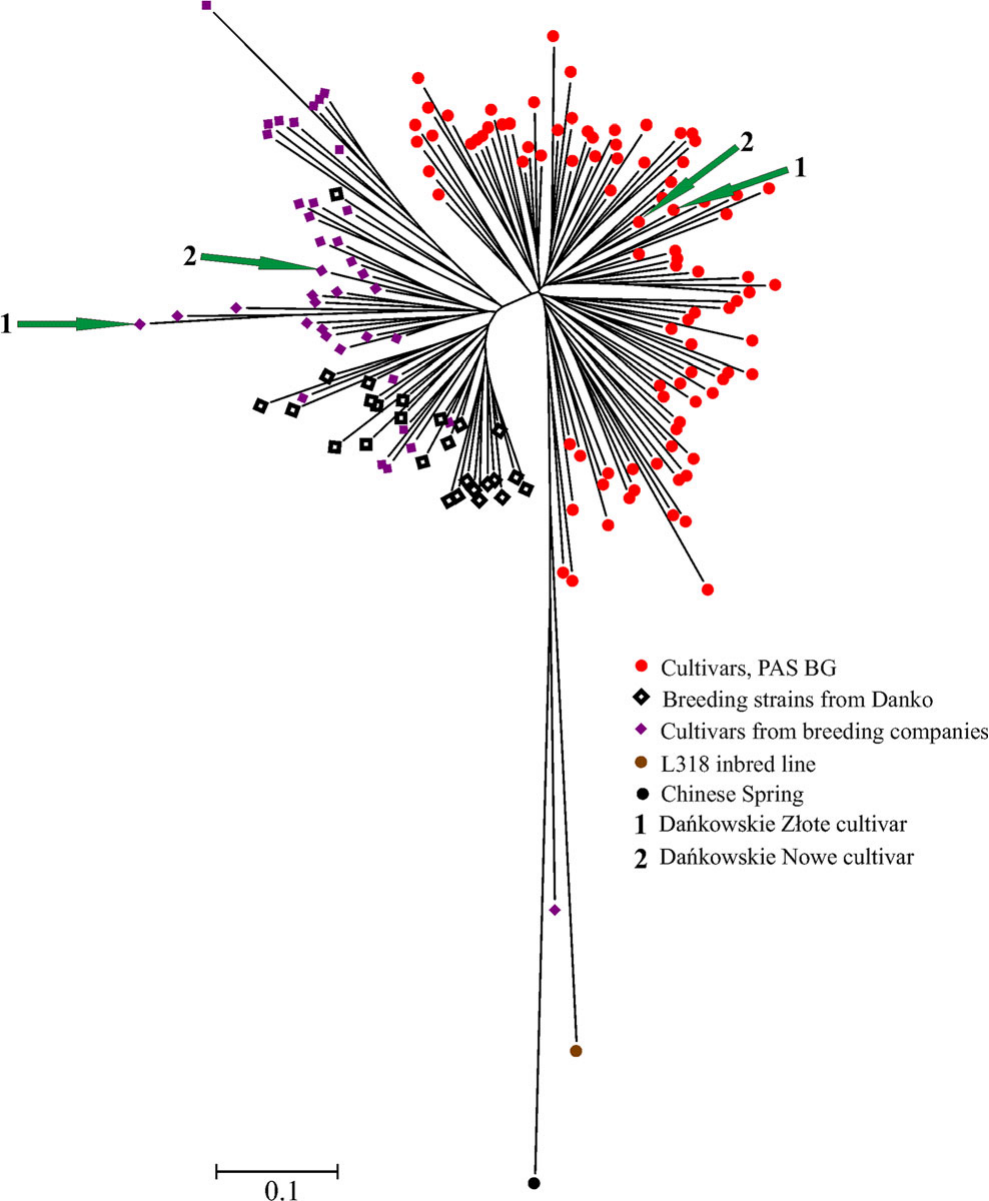
NJ dendrogram showing genetic relationships among 165 rye accessions representing cultivars from gene bank and accessions obtained from breeding companies

**Fig. 7:**
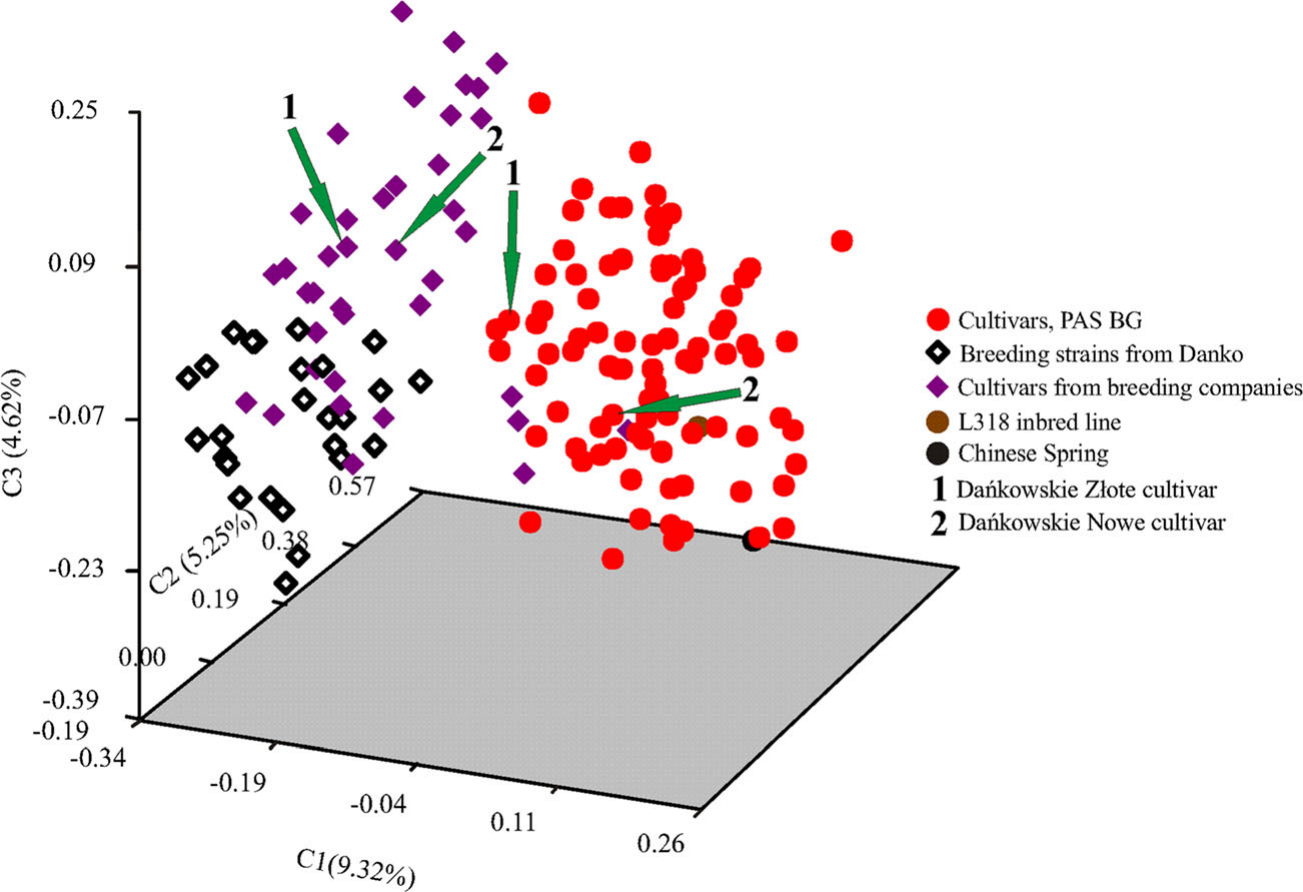
PCoA of 165 rye accessions representing cultivars from gene bank and accessions obtained from breeding companies, based on SSR markers

PCoA comparing cultivars and breeding materials showed clear dissimilarity between accessions from the gene bank and from breeding companies. Two separate clusters were visible both in the dendrogram (Fig. 6) and PCoA graph (Fig. 7). One contained accessions from PAS BG, while accessions from breeding companies were located in the second cluster. Two samples of Dańkowskie Złote and two samples of Dańkowskie Nowe rye cultivars obtained from different sources (one from the Danko company and the second from PAS BG) were not close to each other, occupying separate positions in the PCoA graph and grouped in different clusters in the dendrogram.

In the PCo analysis with labeling according to the assignation to one of the heterotic pools (Petkus or Carsten), the majority of accessions with confirmed Petkus pedigree were closely grouped (Online Resource 6). However, the only accession with confirmed Carsten pedigree was grouped next to accessions derived from Petkus. Contemporary hybrid (F_1_) cultivars were dispersed throughout the PCoA plot.

### Analysis of Molecular Variance

AMOVA analysis of the two model-based populations indicated by STRUCTURE showed that 86 % of the variation was due to differences within populations, while 14 % was due to differences between populations (*P* < 0.001). The pairwise PhiPT value (which is an analogue of F_ST_ in estimation of genetic differentiation) was 0.137 and indicated relatively high degree of differentiation between populations P1 and P2. Much greater proportion of variation within than among populations was found for groups of accessions from different sources and with different improvement status: 87 and 13 %, respectively (*P* < 0.001). When accessions were grouped based on geographical origin, these values were 92 and 8 % (*P* < 0.001). Pairwise PhiPT values estimated between groups of accessions with different source and improvement status indicated a wide range of genetic differentiation between them ranging from low (0.073) between cultivated materials and landraces from PAS BG to very high (0.275) between breeding strains from Danko and accessions from the A. Lukaszewski collection. In the case of plants grouped according to geographical origin, very low 0.003 PhiPT, indicating little genetic differentiation, was detected between accessions from South America and Northern Europe. The highest genetic differentiation (PhiPT = 0.217) was found between accessions from Western Asia and Central Europe (Online Resource 7).

### Core Collection Sampling

**Fig. 8:**
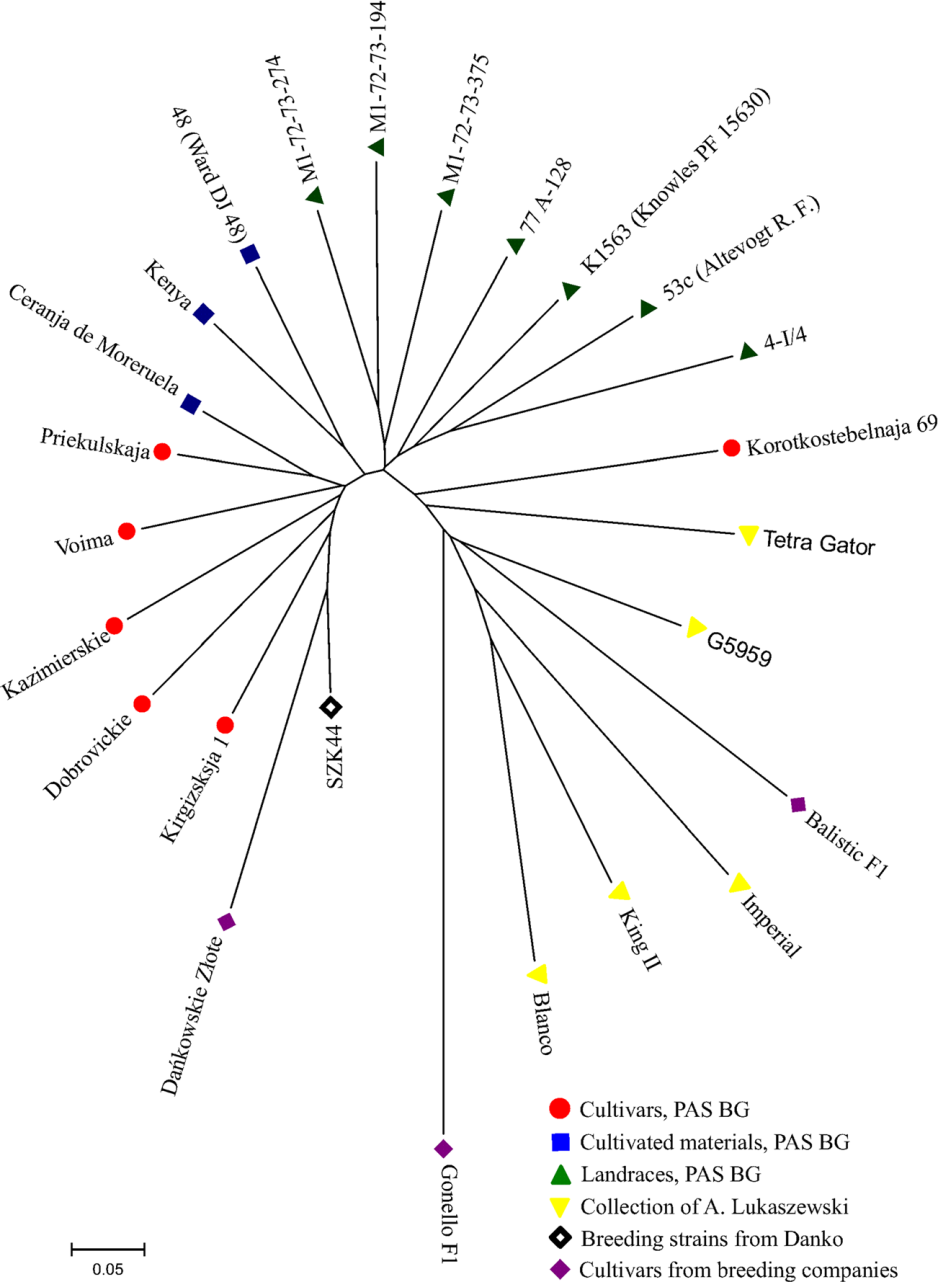
NJ dendrogram showing relationships between rye accessions from the core collection

Out of 367 rye accessions, 25 (6.8 %) were selected as core entries. Core collection entries represented all sources and geographical origins (Table 3). These accessions comprised 93.7 % of all alleles detected within the whole population. The GS value of the core collection ranged from 0.267 to 0.818 (average 0.585). The lowest GS was observed between cultivar Gonello F1 from KWS Lochow and Turkish landrace M1-72-73-194 from PAS BG. The highest GS was found between cultivated material Ceranja de Moreruela from Spain and Lithuanian cultivar Priekulskaja from PAS BG, and these accessions were clustered the closest to each other in the NJ dendrogram (Fig. 8).

**Table 3:** Accessions selected as core collection entries

No.	Accession ID	Accession name	Accession source	Geographical origin
1	SZK44	SZK44	Breeding Strains from Danko	Central Europe
2	OH001	G5959	Collection of A. Lukaszewski	North America
3	OH006	Tetra Gator	Collection of A. Lukaszewski	North America
4	OH008	Imperial	Collection of A. Lukaszewski	North America
5	OH009	King II	Collection of A. Lukaszewski	Northern Europe
6	OH010	Blanco	Collection of A. Lukaszewski	South America
7	OD001	Dańkowskie Złote	Cultivars from breeding companies	Central Europe
8	OL001	Balistic F1	Cultivars from breeding companies	Western Europe
9	OL004	Gonello F1	Cultivars from breeding companies	Western Europe
10	OP010	Dobrovickie	Cultivars PAS BG	Central Europe
11	OP018	Kazimierskie	Cultivars PAS BG	Central Europe
12	OP028	Priekulskaja	Cultivars PAS BG	Eastern Europe
13	OP081	Korotkostebelnaja 69	Cultivars PAS BG	Eastern Europe
14	OP088	Kirgizsksja 1	Cultivars PAS BG	Central Asia
15	OP090	Voima	Cultivars PAS BG	Northern Europe
16	CMP005	Ceranja de Moreruela	Cultivated materials BG PAS	Southern Europe
17	CMP040	Kenya	Cultivated materials BG PAS	Eastern Africa
18	CMP044	48 (Ward DJ 48)	Cultivated materials BG PAS	South America
19	LRC028	K1563 (Knowles PF 15630)	Landraces BG PAS	Eastern Asia
20	LRC037	53c (Altevogt R.F.)	Landraces BG PAS	Southern Asia
21	LRC040	4-I/4	Landraces BG PAS	Balkans
22	LRC101	M1-72-73-375	Landraces BG PAS	Western Asia
23	LRC109	77 A-128	Landraces BG PAS	Southern Europe
24	LRC126	M1-72-73-274	Landraces BG PAS	Western Asia
25	LRC131	M1-72-73-194	Landraces BG PAS	Western Asia

## Discussion

In this study, the genetic diversity within *S. cereale* ssp. *cereale* was thoroughly evaluated using SSR markers. The panel of 367 accessions studied included contemporary and historical cultivars, landraces, and breeding materials from various geographical origins, as well as from various sources and represented the major portion of the inter-species genetic diversity. Therefore, it was possible, to determine whether factors such as geographical origin, source of seeds, and improvement status influence the genetic diversity. The size and variability of this collection helped us to obtain a clearer picture of the rye population structure. Moreover, the SSR markers employed in this study were derived from all rye chromosomes and represented the whole genome. Previous phylogenetic studies on rye have examined far fewer accessions, e.g., 42 in Ma et al. (2004), 47 in Shang et al. (2006), and 114 in Chikmawati et al. (2012). So far, the only other study of equivalent scale to analyze the genetic diversity of the genus *Secale* is that of Bolibok-Brągoszewska et al. (2014), which included 362 of the 367 accessions used here but employed DArT markers. Although, there is the possibility of obtaining a wide genome coverage with DArT markers, their dominant biallelic nature limits the range of the analysis to some extent. The allelic diversity is less informative using these markers than it would be with multi-allelic SSRs (White et al. 2008). In the case of a highly open-pollinated, highly heterozygotic and heterogenic species like rye, SSRs are considered to be the superior marker system for examining genetic diversity. A disadvantage of SSRs is their low throughput, especially in comparison with DArT markers. On the other hand, SSRs are highly accessible because of the relatively simple and cheap methodology, which does not require specialized or sophisticated equipment (Rakoczy-Trojanowska and Bolibok 2004).

In general, the presented results were largely consistent with those of Bolibok-Brągoszewska et al. (2014). In the DArT-based study, 379 accessions were analyzed: 362 cultivated rye accessions, which were used also in the present study, and 17 wild *Secale* accessions that were not analyzed using SSR markers. In the case of cultivated rye accessions, the accessions assigned based on SSR analyses to one of the model-based populations P1 and P2 were largely the same as accessions from populations P3 and P1, respectively, indicated in the DArT-based study (Bolibok-Brągoszewska et al. 2014). The wild accessions were classified mostly as P2 in the DArT-based study. Moreover, PCoA and NJ clustering in both works demonstrated that source of seeds and improvement status contributed significantly to the structure observed in the analyzed set of *S. cereale* accessions. We revealed also a relatively limited diversity in improved rye accessions, both historical and contemporary, as well as lack of correlation between clustering of improved accessions and geographic origin, suggesting common genetic background of rye accessions from diverse geographic regions and extensive germplasm exchange. The resolution of the obtained results was also comparable. The mean GS values calculated in both studies for accession groups formed based on the source of seeds and improvement status almost identical (data not reported).

In our study, we did not analyze the genetic diversity of rye accessions on a single plant basis. Instead a bulking strategy with 96 plants for each accession pooled into one sample was applied. In consequence of DNA pooling some information is lost, for example, it is not possible to estimate allele frequencies within individual accessions. While this limitation is true for both SSR markers used in the present study and for DArT markers applied earlier (Bolibok-Brągoszewska et al 2014), there are other methods, such as genotyping-by-sequencing (Davey et al. 2011), which allow for estimation of allele frequencies in pooled samples. Therefore, it will be possible to obtain detailed information on within-accession diversity in the future, using DNA samples prepared for the present study. The multi-allelic nature of SSR markers was an important feature in the analyses of pooled samples. While in the case of DArT markers (Bolibok-Brągoszewska et al 2014), a score of one (1) or zero (0) was obtained for each accession, in the case of SSR markers we observed an allele (or in most cases several alleles), that could be defined and distinguished by its length. Furthermore, in the case of accessions groups of different origin on improvements status, we were able to identify frequent or unique SSR alleles (Table 2) or number of alleles detected using a given SSR marker. While in the case of DArT markers, it was only possible to calculate the percentage of 1 or 0 score within a given accession subgroup. Thus, in our opinion, after performing SSR assays, we obtained a fuller picture of genetic diversity in the collection.

There are many examples in the literature of the use of SSR markers in assessing genetic diversity of plant species. Usually, a relatively low number of SSRs is sufficient to accurately reflect genetic structure and diversity among a high number of accessions. For example, 23 SSRs were used in genetic diversity studies of 3342 cucumber accessions (Lv et al. 2012), while 48 markers were used to examine 2945 accessions of chickpea (Upadhyaya et al. 2008). So far, there have been only a few examples where SSR markers have been utilized in studies of genetic diversity in rye. In the study of Shang et al. (2006), the genetic diversity and phylogenic relationships among 30 *Secale* accessions and 47 cultivated ryes were evaluated using 24 *S. cereale* microsatellite markers. Bolibok et al. (2005) utilized 38 SSR markers to assess the genetic diversity of 30 rye inbred lines. Thirteen SSR markers were used in the work of Myśków et al. (2010) to conduct genetic diversity analysis on 48 rye inbred lines. In comparison, the present study used 22 polymorphic SSRs, representing different rye chromosomes, in the phylogenetic analysis of 367 accessions.

The PIC value calculated for SSR markers used for genotyping was relatively high with an average of 0.57 (range 0.18–0.93) which indicates their high informativeness. The average percentage of polymorphic alleles for the set of 22 markers was also high (93 %). This was predictable because the public markers were not selected randomly, but based on the results of analyses using six diverse rye accessions and 42 SSR markers. The high values of the marker informativeness parameters and the congruency of the results reported here with the results obtained based on over 1000 genome-wide DArT markers (Bolibok-Brągoszewska et al. 2014) indicate that the selected 22 SSR markers are well suited for assessment of genetic diversity patterns in rye germplasm.

We used the following methods to demonstrate genetic relationships between the analyzed rye accessions: NJ cluster analysis, PCoA, and Bayesian clustering. The results obtained with all three methods were generally consistent. NJ clustering and PCoA distinguished two major groups within the analyzed accessions, dividing them according to the source of seeds and improvement status, and revealed that cultivars from breeding companies were clearly distinct from those reproduced in gene bank. In particular, cultivars and breeding strains from Polish and German breeding companies clustered in one group, and were distinct from all other accessions. The separation of currently cultivated accessions may be the result of adaptation of modern cultivars to the requirements of agriculture and environmental conditions in Central and Northern Europe. However, it is more likely that the different methods of plant material reproduction used in breeding companies and gene banks have affected the genetic diversity of accessions from these two sources. It is noteworthy that even samples of the same rye cultivar from different sources of seeds occupied two different positions in both the PCoA graph and the dendrogram (e.g., two samples of Dańkowskie Złote and two of Dańkowskie Nowe, one sample obtained directly from the company Danko and the second from PAS BG). While this result could be attributed to a mistake in a labeling of seed samples, an alternative explanation may be found in the work of Chebotar et al. (2003), where it was suggested that for open-pollinated species, the genetic integrity of an accession may be changed in each regeneration cycle, which can alter the SSR pattern. Genetic changes in seeds of the Dańkowskie Złote rye cultivar induced by long-term storage effects and consecutive regeneration cycles were also identified by the use of AFLP markers (Chwedorzewska et al. 2002) and SSR markers (Boczkowska and Puchalski 2012). Surprising results were obtained also for Gonello F_1_ variety, which turned out to be distinct from other hybrid varieties form the same breeding company (KWS Lochow) and also from population and hybrid varieties from other breeding companies. Similar results for Gonello were obtained earlier using DArT markers (Bolibok-Brągoszewska et al. 2014). As we mentioned previously (Bolibok-Brągoszewska et al. 2014), the pedigree information does not provide support for the observed distinctness of Gonello; hence, further analyses of additional independent samples would be needed to elucidate this problem.

As discussed above, factors such as reproduction or the manner of storage of plant material can greatly influence genetic variation. This may reflect differences in the procedures of plant reproduction employed by gene banks and breeding companies. In the case of open-pollinated plants, it is important to consider regeneration management in ex situ collections, particularly the optimal population size and distance between regeneration plots (Börner et al. 2012). It may be concluded that the lack of a standardized procedure for plant reproduction may have caused variation in the genetic diversity of the same rye accessions obtained from different institutions.

All our analyses demonstrated the high level of similarity between currently cultivated rye forms and breeding materials. Dendrograms and PCoA plots clearly showed that Polish and German accessions, in particular, grouped close together. STRUCTURE analysis assigned strains from Danko and modern cultivars to one model-based population (P1). Genetic similarity within cultivars from breeding companies and within breeding strains from Danko was relatively high. It is also noteworthy that no unique alleles were observed in accessions obtained from Danko and the values for mean diversity (*h* = 0.113) and unbiased diversity (uh = 0.118) were the lowest in this germplasm group. Evidence indicating low genetic diversity in Polish rye was previously obtained by Rafalski et al. (2002) who analyzed five open-pollinated cultivars. The low diversity level suggested that breeding programs of open-pollinated cultivars had reached their limit of genetic variability (Rafalski et al. 2002). Similarly, in the study of Shang et al. (2006), the level of genetic similarity (GS) in cultivated rye from different parts of the world was found to be insignificant. An AFLP-based study involving a broader range of plant material indicated that the genetic diversity of rye in Europe tended to decrease when moving from East to North (Chikmawati et al. 2012), with the lowest genetic differentiation and genetic distance found between accessions from Eastern and Central Europe. These findings suggest that genetic variation between contemporary cultivars has been reduced during the breeding process. The reason for this is that the majority of rye breeding programs focus on common aims, with the main target traits, besides grain yield, being tolerance to abiotic stresses, performance at high plant density, good kernel development, and resistance to powdery mildew or stem rust. As a consequence, this causes uniformity and increased homogeneity in commercial cultivars (Chikmawati et al. 2012; Persson and von Bothmer 2000). Additionally, the majority of the open-pollinated rye forms currently cultivated in Western and Central Europe have a common origin and are directly descended from the Petkus population (Fischer et al. 2010). Also in the present study, the contemporary cultivars, which were derived from Petkus heterozygotic pool (according to the obtained pedigree information) and originated mainly from Poland and Germany, were closely grouped in the PCoA plot (Online Resource 7). Moreover, most of them (82.5 %) was assigned to the same model-based population (P1) by STRUCTURE analysis. Worth to point out is also the fact that the accession derived from Carsten heterotic pool was also assigned to P1 model-based population and was grouped next to accessions derived from Petkus in the PCoA plot. However, the available data is not sufficient for a thorough discussion of this issue. Information on genetic background was not accessible for many cultivars, and based on the obtained information, only one accession representing Carsten genepool was included in the study. Nevertheless, obtained results confirm the common genetic background of contemporary cultivars and suggest extensive germplasm exchange.

The current breeding practices result in a decrease in genetic distance and can lead to a reduction in heterozygosity (Falconer and Mackay 1996). This causes a narrowing of the genetic pool of rye cultivars, which is highly undesirable since it can result in the loss of significant features like resistance to different stresses and nutritional value. Such plants may also have problems in adapting to changing environmental conditions. The introduction of landraces into rye breeding programs could extend the genetic pool of currently cultivated accessions. Landraces are early, highly heterogeneous forms of cultivars that were selected for use in subsistence agriculture, so they give a relatively low but stable yield (McCouch 2004). Our results show that the 155 landraces included in the analysis constituted the most divergent group, with the highest within-group variance. Similar results were obtained by Bolibok-Brągoszewska et al. (2014) using DArT markers. Landraces displayed considerable diversity and were distant from accessions obtained from breeders. This finding is not surprising because landraces are closely related to the wild ancestors of rye and embody much more variation than modern high-yielding cultivars (McCouch 2004), and it has been postulated that landraces could represent a valuable source of genetic variation that has been lost in modern breeding (Gailîte et al. 2013; Boczkowska and Traczyk 2013). Landraces may be a source of potentially useful alleles also for rye breeding. The results of Falke et al. (2008) indicated that exotic genetic resources like landraces in rye carry favorable alleles for baking quality traits, which may be exploited for improving elite breeding material by marker-assisted selection (MAS). Nevertheless, breeders are reluctant to introduce genetically distinct accessions like landraces into breeding programs, fearing that some of the introduced alleles may negatively influence breeding values. Thus, the results of the present study could be used to facilitate the material selection for broadening the genetic diversity within heterotic pools and promote the exploitation of less adapted germplasm in breeding programs.

In general, the clustering of rye accessions was more weakly correlated with geographic origin than with the source of seeds. However, some accessions grouped according to their geographical origin could be distinguished in the dendrogram and PCoA plot. Similar results were obtained using DArT markers (Bolibok-Brągoszewska et al. 2014). Weak correlations between genetic diversity in rye and geographical origin were identified by Chikmawati et al. (2012), Shang et al. (2006), and Persson and von Bothmer (2000) through analyses of AFLPs, SSRs, and organellar sequences, respectively. In the present study, accessions from Central Europe displayed the most visible genetic separation. However, this observation may result from the fact that the majority of Central European accessions were obtained directly from breeding companies in which the same selection criteria and breeding methods are applied.

It was shown in several studies on genetic diversity that growth habit (winter vs. spring) is one of the major determinants of the population structure in cereals (Ma et al. 2004; Alheit et al. 2012; Wang et al. 2012). Unfortunately, we were unable to check how this factor influences the genetic diversity structure in rye, since only one variety from our germplasm set represented spring type (Bojko), and the information on growth habit of landraces was not available. Nevertheless, Bojko variety grouped closely with other accessions obtained from breeding companies.

Another goal of our study was the selection of accessions to form a core collection. Core collections are a set of accessions derived from an existing collection that are selected to represent the widest possible spectrum of genetic variation in a given population in order to minimize the cost of genetic conservation (Brown 1989). The selected core collection constituted 6.8 % of all rye accessions and involved 93.7 % of SSR alleles that have been identified. The rye core entries were selected from a higher number of accessions than used in any previous study. A limited number of accessions, characterized by high genetic variation, represent a useful tool in the study of diversity within a population. Core collections play a very important role, especially in gene banks which face significant problems connected with the size and organization of plant germplasm collections. Nowadays, over 80 rye germplasm collections are maintained and the total number of accessions is estimated to be over 21 000 (FAO 2010). The proposed core collection could be the first step to simplify access to genetic diversity contained in rye germplasm and to enable its efficient use in basic and applied research. Moreover, our core collection could be treated as a testing panel in evaluating newly developed genetic markers or in studies on sequence diversity of selected genome fragments.

## Conclusions

In the presented study, we conducted a very large and comprehensive study of genetic diversity in *S. cereale* species. As a result, we revealed a relatively limited genetic diversity within contemporary rye cultivars and breeding strains, indicating the common genetic background and germplasm exchange. We found also a lack of correlation between clustering of rye accessions, including improved cultivars, and their geographic origin. Results obtained in the presented work highlighted the need for broadening the genetic diversity in breeding programs.

We also defined a core collection of rye accession, which represented the vast majority of the observed genetic diversity. The proposed core collection could contribute to simplification of germplasm maintenance issues in ex situ collections, as well as find practical use in basic research involving testing of newly developed markers and analyses of sequence diversity of selected genes/gene fragments.

Obtained data confirmed also that the set of 22 SSR markers used in the study revealed a realistic picture of genetic diversity between 367 rye accessions. Therefore, it can be recommended for further germplasm characterization project.

Results obtained in our study could be also helpful for breeders considering introduction of less adapted germplasm to breeding programs.

## Electronic Supplementary Material

Online Resource 1 367 *Secale cereale* accessions used in the genetic diversity studies. (PDF 369 kb)

Online Resource 2 SSR markers used in preliminary analyses. (PDF 83 kb)

Online Resource 3 The result of Mantel test showing a strong positive correlation between Jaccard and Dice coefficient. (PDF 541 kb)

Online Resource 4 GS values (according to the Jaccard coefficient) for pairs of accessions. (PDF 6710 kb)

Online Resource 5 Delta K values (number of populations assumed) ranging from 1 to 20. (PDF 1347 kb)

Online Resource 6 Results of Analysis of Molecular Variance. (PDF 34 kb)

Online Resource 7 PCoA of 367 rye accessions based on 22 SSR markers. Accessions labeled according to their pedigree. (PDF 1369 kb)

## References

[CR1] Alheit KV, Maurer HP, Reif JC, Tucker MR, Hahn V, Weissmann EA, Würschum T (2012). Genome-wide evaluation of genetic diversity and linkage disequilibrium in winter and spring triticale (× Triticosecale Wittmack). BMC Genomics.

[CR2] Andersson R, Fransson G, Tietjen M, Åman P (2009). Content and molecular-weight distribution of dietary fiber components in wholegrain rye flour and bread. J Agric Food Chem.

[CR3] Benbouza H, Jacquemin JM, Baudoin JP, Mergeai B (2006). Optimization of a reliable, fast, cheap and sensitive silver staining method to detect SSR markers in polyacrylamide gels. Biotechnol Agron Soc Environ.

[CR4] Boczkowska M, Puchalski J (2012). SSR studies of genetic changes in relation to long-term storage and field regeneration of rye (*Secale cereale* L) seeds. Seed Sci Technol.

[CR5] Boczkowska M, Traczyk E (2013). Genetic diversity among Polish landraces of common oat (*Avena sativa* L). Genet Resour Crop Evol.

[CR6] Bolibok H, Rakoczy-Trojanowska M, Hromada A, Pietrzykowski R (2005). Efficiency of different PCR-based marker systems in assessing genetic diversity among winter rye (*Secale cereale* L) inbred lines. Euphytica.

[CR7] Bolibok-Brągoszewska H, Heller-Uszyńska K, Wenzl P, Uszyński G, Kilian A, Rakoczy-Trojanowska M (2009). DArT markers for the rye genome - genetic diversity and mapping. BMC Genomics.

[CR8] Bolibok-Brągoszewska H, Targońska M, Bolibok L, Kilian A, Rakoczy-Trojanowska M (2014). Genome-wide characterization of genetic diversity and population structure in *Secale*. BMC Plant Biol.

[CR9] Börner A, Khlestkina EK, Chebotar S, Nagel M, Arif MAR, Neumann K, Kobiljski B, Lohwasser U, Röder MS (2012). Molecular markers in management of ex situ PGR – a case study. J Biosci.

[CR10] Brown AHD (1989). Core collections—a practical approach to genetic-resources management. Genome.

[CR11] Burger JC, Lee S, Ellstrand NC (2006). Origin and genetic structure of feral rye in the western United States. Mol Ecol.

[CR12] Chebotar S, Röder MS, Korzun V, Saal B, Weber WE, Börner A (2003). Molecular studies on genetic integrity of open pollinating species rye (*Secale cereale* L.) after long term genebank maintenance. Theor Appl Genet.

[CR13] Chikmawati T, Skovmand B, Gustafson JP (2005). Phylogenetic relationships among *Secale* species revealed by amplified fragment length polymorphisms. Genome.

[CR14] Chikmawati T, Miftahudin M, Skovmand B, Gustafson JP (2012). Amplified fragment length polymorphism-based genetic diversity among cultivated and weedy rye (*Secale cereale* L) accessions. Genet Resour Crop Evol.

[CR15] Chwedorzewska KJ, Bednarek PT, Puchalski J, Krajewski P (2002). AFLP-profiling of long-term stored and regenerated rye genebank samples. Cell Mol Biol Lett.

[CR16] Davey JW, Hohenlohe PA, Etter PD, Boone JQ, Catchen JM, Blaxter ML (2011). Genome-wide genetic marker discovery and genotyping using next-generation sequencing. Nat Rev Genet.

[CR17] Earl DA, von Holdt BM (2011). STRUCTURE HARVESTER: a website and program for visualizing STRUCTURE output and implementing the Evanno method. Conserv Genet Resour.

[CR18] Evanno G, Regnaut S, Goudet J (2005). Detecting the number of clusters of individuals using the software STRUCTURE: a simulation study. Mol Ecol.

[CR19] Falconer DS, Mackay TFC (1996) Introduction to quantitative genetics. Longman, Harlow

[CR20] Falke KC, Hackauf B, Korzun V, Schondelmaier J, Wilde P, Wehling P, Wortmann H, Mank R, van der Voort JR, Maurer HP, Miedaner T, Geiger HH, Sušić Z (2008). Establishment of introgression libraries in hybrid rye (*Secale cereale* L.) from an Iranian primitive accession as a new tool for rye breeding and genomics. Theor Appl Genet.

[CR21] FAO (2010). The second report on the state of the world’s plant genetic resources for food and agriculture.

[CR22] Fischer S, Melchinger AE, Korzun V, Wilde P, Schmiedchen B, Möhring J, Piepho HP, Dhillon BS, Würschum T, Reif JC (2010). Molecular marker assisted broadening of the Central European heterotic groups in rye with Eastern European germplasm. Theor Appl Genet.

[CR23] Fu S, Tang Z, Ren Z, Zhang H, Yan B (2010). Isolation of rye-specific DNA fragment and genetic diversity analysis of rye genus *Secale* L using wheat SSR markers. J Genet.

[CR24] Gailîte A, Gaile A, Gaile I, Voronova A, Veinberga I, Kokare A, Ruòìis DA (2013). Genotypic assessment of the Latvian rye (*Secale cereale* L.) collection. Proc Latvian Acad Sci Sect B.

[CR25] Geiger HH, Miedaner T, Coors JG, Pandey S (1999). Hybrid rye and heterosis. Genetics and exploitation of heterosis in crops.

[CR26] Hackauf B, Wehling P (2002). Identification of microsatellite polymorphisms in expressed portion of the rye genome. Plant Breed.

[CR27] Hackauf B, Rudd S, Voort J, Miedaner T, Wehling P (2009). Comparative mapping of DNA sequences in rye (*Secale cereale* L) in relation to the rice genome. Theor Appl Genet.

[CR28] Haseneyer G, Schmutzer T, Seidel M, Zhou R, Mascher M, Schön CC, Taudien S, Scholz U, Stein N, Mayer K, Bauer E (2011). From RNA-seq to large-scale genotyping - genomics resources for rye (*Secale cereale* L). BMC Plant Biol.

[CR29] Hurtado P, Olsen KM, Buitrago C, Ospina C, Marin J, Duque M, de Vicente C, Wongtiem P, Wenzel P, Killian A, Adeleke M, Fregene M (2008). Comparison of simple sequence repeat (SSR) and diversity array technology (DArT) markers for assessing genetic diversity in cassava (*Manihot esculenta* Crantz). Plant Genet Resour Character Util.

[CR30] Isik Z, Parmaksiz I, Coruh C, Geylan-Su YS, Cebeci O, Beecher B, Budak H (2007). Organellar genome analysis of rye (*Secale cereale*) representing diverse geographic regions. Genome.

[CR31] Jakobsson M, Rosenberg NA (2007). CLUMPP: a cluster matching and permutation program for dealing with label switching and multimodality in analysis of population structure. Bioinformatics.

[CR32] Kim KW, Chung HK, Cho GT, Ma KH, Chandrabalan D, Gwag JG, Kim TS, Cho EG, Park YJ (2007). PowerCore: a program applying the advanced M strategy with a heuristic search for establishing core sets. Bioinformatics.

[CR33] Korzun V, Malyshev S, Voylokov AV, Borner A (2001). A genetic map of rye (*Secale cereale* L.) combining RFLP, isozyme, protein, microsatellite and gene loci. Theor Appl Genet.

[CR34] Laidò G, Mangini G, Taranto F, Gadaleta A, Blanco A, Cattivelli L, Marone D, Mastrangelo AM, Papa R, De Vita P (2013). Genetic diversity and population structure of tetraploid wheats (*Triticum turgidum* L.) estimated by SSR, DArT and pedigree data. PLoS One.

[CR35] Lv J, Qi J, Shi Q, Shen D, Zhang S, Shao G, Li H, Sun Z, Weng Y, Shang Y, Gu X, Li X, Zhu X, Zhang J, van Treuren R, van Dooijeweert W, Zhang Z, Huangl S (2012). Genetic diversity and population structure of cucumber (*Cucumis sativus* L.). PLoS One.

[CR36] Ma R, Yli-Matilla T, Pulli S (2004). Phylogenetic relationship among genotypes of worldwide collection of spring and winter ryes (*Secale cereale* L.) determined by RAPD-PCR markers. Hereditas.

[CR37] Matos M, Pinto-Carnide O, Benito C (2001). Phylogenetic relationships among Portuguese rye based on isozyme, RAPD and ISSR markers. Hereditas.

[CR38] McCouch S (2004). Diversifying selection in plant breeding. PLoS Biol.

[CR39] Myśków B, Milczarski P, Masojć P (2010). Comparison of RAPD, ISSR and SSR markers in assessing genetic diversity among rye (*Secale cereale* L.) inbred lines. Plant Breed Seed Sci.

[CR40] Peakall R, Smouse PE (2012). GenAlEx 65: genetic analysis in Excel Population genetic software for teaching and research-an update. Bioinformatics.

[CR41] Persson K, von Bothmer R (2000). Assessing the allozyme variation in cultivars and Swedish landraces of rye (*Secale cereale L*.). Hereditas.

[CR42] Pillen K, Binder A, Kreuzkam AB, Ramsay L, Waugh R, Förster J, Léon J (2000). Mapping new EMBL-derived barley microsatellites and their use in differentiating German barley cultivars. Theor Appl Genet.

[CR43] Pritchard JK, Stephens M, Donnelly P (2000). Inference of population structure using multilocus genotype data. Genetics.

[CR44] Rafalski A, Madej L, Wiśniewska I, Gaweł M (2002). The genetic diversity of components of rye hybrids. Cell Mol Biol Lett.

[CR45] Rakoczy-Trojanowska M, Bolibok H (2004). Characteristics and a comparison of three classes of microsatellite-based markers and their application in plants. Cell Mol Biol Lett.

[CR46] Rohlf FJ (2000) NTSYS-pc: numerical taxonomy and multivariate analysis system, version 2.1. Exeter Software, Setauket, New York

[CR47] Rosén LAH, Östman EM, Shewry PR, Ward JL, Andersson AAM, Piironen V, Lampi AM, Rakszegi M, Bedö Z, Björck IME (2011). Postprandial glycemia, insulinemia, and satiety responses in healthy subjects after whole grain rye bread made from different rye varieties 1. J Agric Food Chem.

[CR48] Rosenberg NA (2004). DISTRUCT: a program for the graphical display of population structure. Mol Ecol Notes.

[CR49] Roy JK, Balyan HS, Prasad M, Gupta PK (2002). Use of SAMPL for a study of DNA polymorphism, genetic diversity and possible gene tagging in bread wheat. Theor Appl Genet.

[CR50] Saal B, Wricke G (1999). Development of simple sequence repeat markers in rye (*Secale cereale* L.). Genome.

[CR51] Schlegel R, Melz G (1996). Genetic linkage map of rye (Secale cereale L.). Vortr Pflanzenzuchtg.

[CR52] Sencer HA, Hawkes JG (1980). On the origin of cultivated rye. Biol J Linn Soc.

[CR53] Shang H-Y, Wei Y-M, Wang X-R, Zheng Y-L (2006). Genetic diversity and phylogenetic relationships in the rye genus *Secale* L. (rye) based on *Secale cereale* microsatellite markers. Genet Mol Biol.

[CR54] Simko I, Eujayl I, van Hintum TJ (2012). Empirical evaluation of DArT, SNP, and SSR marker-systems for genotyping, clustering, and assigning sugar beet hybrid varieties into populations. Plant Sci.

[CR55] Skuza L, Rogalska S, Bocianowski J (2007). RFLP analysis of mitochondrial DNA in the genus *Secale*. Acta Biol Cracov Ser Bot.

[CR56] Smith JCS, Duvick DN, Smith OS, Cooper M, Feng L (2004). Changes in pedigree backgrounds of Pioneer brand maize hybrids widely grown from 1930 to 1999. Crop Sci.

[CR57] Tamura K, Peterson D, Peterson N, Stecher G, Nei M (2011). MEGA5: molecular evolutionary genetics analysis using maximum likelihood, evolutionary distance, and maximum parsimony methods. Mol Biol Evol.

[CR58] Upadhyaya HD, Dwivedi SL, Baum M, Varshney RK, Udupa SM, Gowda CLL, Hoisington D, Singh S (2008). Genetic structure, diversity, and allelic richness in composite collection and reference set in chickpea (*Cicer arietinum* L.). BMC Plant Biol.

[CR59] Wang H, Smith KP, Combs E, Blake T, Horsley RD, Muehlbauer GJ (2012). Effect of population size and unbalanced data sets on QTL detection using genome-wide association mapping in barley breeding germplasm. Theor Appl Genet.

[CR60] White J, Law JR, MacKay I, Chalmers KJ, Smith JSC, Kilian A, Powell W (2008). The genetic diversity of UK, US and Australian cultivars of Triticum aestivum measured by DArT markers and considered by genome. Theor Appl Genet.

