# Supplementary material for: Assessment of Genetic Diversity in Secale cereale Based on SSR Markers
Source: Plant Mol Biol Report. 2015 Jun 6;34:37–51. doi: 10.1007/s11105-015-0896-4 (PMC4722074; doi:10.1007/s11105-015-0896-4)
Supplement: Supplementary file 1 — 367 Secale cereale accessions used in the genetic diversity studies. (PDF 369 kb) [file 11105_2015_896_MOESM1_ESM.pdf]

No	Accession code	Introduction number	Accession name	Origin	Improvement status	Geographical region	Source of seeds	Population indicted by STRUCTURE	Heterotic pool
1	OP001	16275/81	ALTOBAR	ESP	Cultivar	Southern Europe	PAS BG	P1/P2	-
2	OP002	18641/83	ANTONINSKIE	POL	Cultivar	Central Europe	PAS BG	P1	-
3	OP003	1356/71	BEAULIEU	FRA	Cultivar	Western Europe	PAS BG	P1	Petkus
4	OP004	43/70	BEZENCUKSKAJA ZELTOZERNAJA	RUS	Cultivar	Eastern Europe	PAS BG	P1	-
5	OP005	1357/71	BJORN	SWE	Cultivar	Northern Europe	PAS BG	P1	Petkus
6	OP006	6970/76	BONEL	USA	Cultivar	North America	PAS BG	P1	-
7	OP007	14955/80	CELESTYNER	BEL	Cultivar	Western Europe	PAS BG	P1	-
8	OP008	2384/73	HAR'KOVSKAJA 194	UKR	Cultivar	Eastern Europe	PAS BG	P1/P2	-
9	OP009	1274/71	DANAE	DEU	Cultivar	Western Europe	PAS BG	P1	-
10	OP010	5781/75	DOBROVICKIE	CSK	Cultivar	Central Europe	PAS BG	P1	-
11	OP011	2521/73	DOMINANT	NLD	Cultivar	Western Europe	PAS BG	P1	-
12	OP012	7020/76	ELBON	USA	Cultivar	North America	PAS BG	P1/P2	-
13	OP013	7031/76	EVEREST	FRA	Cultivar	Western Europe	PAS BG	P1/P2	-
14	OP014	18640/83	GRANUM	BLR	Cultivar	Eastern Europe	PAS BG	P1/P2	-
15	OP015	1411/71	GUARDA	PRT	Cultivar	Southern Europe	PAS BG	P1	-
16	OP016	8828/79	HARU 4	JPN	Cultivar	Western Asia	PAS BG	P1/P2	-
17	OP017	2681/73	KAMALINSKAJA 13	RUS	Cultivar	Eastern Europe	PAS BG	P1/P2	-
18	OP018	18636/83	KAZIMIERSKIE	POL	Cultivar	Central Europe	PAS BG	P1/P2	-
19	OP019	2376/73	KISVARDAI	HUN	Cultivar	Central Europe	PAS BG	P1	-
20	OP020	2381/73	KOLAROVKA	BGR	Cultivar	Balkans	PAS BG	P1	-
21	OP021	5805	KUNGS	SWE	Cultivar	Northern Europe	PAS BG	P1	Petkus
22	OP022	1368/71	MIKULICKIE WCZESNE	POL	Cultivar	Central Europe	PAS BG	P1	-
23	OP023	580/75	NOVOZYBKOVSKAJA 4	RUS	Cultivar	Eastern Europe	PAS BG	P2	-
24	OP024	2685/73	OMKA	RUS	Cultivar	Eastern Europe	PAS BG	P2	-
25	OP025	2361/73	OVARI	HUN	Cultivar	Central Europe	PAS BG	P1	-
26	OP026	7217/76	PETKA	DEU	Cultivar	Western Europe	PAS BG	P1	Petkus
27	OP027	7221/76	PETKUS	DEU	Cultivar	Western Europe	PAS BG	P1	Petkus
28	OP028	44/70	PRIEKULSKAJA	LVA	Cultivar	Eastern Europe	PAS BG	P1	-
29	OP029	2438/73	PULAWSKIE PASTEWNE	POL	Cultivar	Central Europe	PAS BG	P1/P2	Petkus
30	OP030	5684/75	RADOSINSKIE REKORD	SVK	Cultivar	Central Europe	PAS BG	P1/P2	Petkus
31	OP031	2689	RZHAKSINSKAJA	RUS	Cultivar	Eastern Europe	PAS BG	P1/P2	Petkus
32	OP032	5011/75	SADOVA	BGR	Cultivar	Balkans	PAS BG	P1	-
33	OP033	55/70	STENDSKAJA II	LVA	Cultivar	Eastern Europe	PAS BG	P1/P2	-
34	OP034	2694	TARASHHANSKAJA 2	RUS	Cultivar	Eastern Europe	PAS BG	P1	-
35	OP035	1335/71	TOIVO	FIN	Cultivar	Northern Europe	PAS BG	P1	-
36	OP036	2965	UDINSKAJA	RUS	Cultivar	Eastern Europe	PAS BG	P1/P2	-
37	OP037	18642/83	WIERZBIENSKIE	POL	Cultivar	Central Europe	PAS BG	P1	-
38	OP038	8811/79	ANIMO	NLD	Cultivar	Western Europe	PAS BG	P1	Petkus
39	OP039	1332/71	CESKE	CSK	Cultivar	Central Europe	PAS BG	P1	-
40	OP040	47/70	HAR'KOVSKAJA 60	UKR	Cultivar	Eastern Europe	PAS BG	P1	-
41	OP041	2394/73	CHROBRE	POL	Cultivar	Central Europe	PAS BG	P1	-
42	OP042	2679/73	DRUZBA	BLR	Cultivar	Eastern Europe	PAS BG	P1	-
43	OP043	2547/73	DANKOWSKIE NOWE	POL	Cultivar	Central Europe	PAS BG	P1	Petkus
44	OP044	2518/73	DANKOWSKIE SREBRNE	POL	Cultivar	Central Europe	PAS BG	P1	Petkus
45	OP045	1595/72	DANKOWSKIE ZIELONKOWE	POL	Cultivar	Central Europe	PAS BG	P1/P2	Petkus
46	OP046	2651/73	DANKOWSKIE ZLOTE	POL	Cultivar	Central Europe	PAS BG	P1	Petkus
47	OP047	7909/77	IMPERIAL RYE	CAN	Cultivar	North America	PAS BG	P1	Petkus
48	OP048	7169/76	KUSTRO	DEU	Cultivar	Western Europe	PAS BG	P1	Petkus
49	OP049	1294/71	LITOVSKAJA	LTU	Cultivar	Eastern Europe	PAS BG	P1/P2	-
50	OP050	1372/71	PANCERNE	POL	Cultivar	Central Europe	PAS BG	P1	Petkus
51	OP051	6158/75	PULAWSKIE ZIELONKOWE	POL	Cultivar	Central Europe	PAS BG	P2	Petkus
52	OP052	2696/73	URALSKAJA	RUS	Cultivar	Eastern Europe	PAS BG	P2	-
53	OP053	6168/75	WIELKOPOLSKIE	POL	Cultivar	Central Europe	PAS BG	P1	Petkus

54	OP054	1370/71	WLOSZANOWSKIE	POL	Cultivar	Central Europe	PAS BG	P1	Petkus
55	OP055	2697/73	VOLZHANKA	RUS	Cultivar	Eastern Europe	PAS BG	P1/P2	-
56	OP056	6178	ZELANDZKIE	POL	Cultivar	Central Europe	PAS BG	P1	Petkus
57	OP057	5839	ZIMA	RUS	Cultivar	Eastern Europe	PAS BG	P2	-
58	OP058	2699/73	ZHYTOMIRSKAJA	UKR	Cultivar	Eastern Europe	PAS BG	P1	-
59	OP059	828/96	KORMOVAJA 61	RUS	Cultivar	Eastern Europe	PAS BG	P2	-
60	OP060	8409/78	FORRAJERO MASSAUX	ARG	Cultivar	South America	PAS BG	P1/P2	-
61	OP061	1367/71	GARCZYNSKIE	POL	Cultivar	Central Europe	PAS BG	P1	Petkus
62	OP062	16103	PANICENI	ROM	Cultivar	Eastern Europe	PAS BG	P1	-
63	OP063	8413/78	PICO M.A.G.	ARG	Cultivar	South America	PAS BG	P1/P2	-
64	OP064	2687/73	POLESSKAJA	UKR	Cultivar	Eastern Europe	PAS BG	P1	-
65	OP065	1369/71	SMOLICKIE	POL	Cultivar	Central Europe	PAS BG	P1	Petkus
66	OP066	2400/73	WOJCIESZYCKIE	POL	Cultivar	Central Europe	PAS BG	P1	Petkus
67	OP067	7395/76	WRENS ABRUZZI	USA	Cultivar	North America	PAS BG	P1/P2	-
68	OP068	17533	YAN AN	CHN	Cultivar	Western Asia	PAS BG	P2	-
69	OP069	39/70	LISICYNA; LISYCYNA	RUS	Cultivar	Eastern Europe	PAS BG	P2	-
70	OP070	52/70	BALTIJA	LTU	Cultivar	Eastern Europe	PAS BG	P1/P2	-
71	OP071	7475/76	CADI	CHE	Cultivar	Western Europe	PAS BG	P1	Petkus
72	OP072	7032/76	EXPLORER	USA	Cultivar	North America	PAS BG	P1/P2	-
73	OP073	7038/76	FLORIDA BLACK	USA	Cultivar	North America	PAS BG	P1/P2	-
74	OP074	6963/76	ANTELOPE	AUT	Cultivar	Western Europe	PAS BG	P1	-
75	OP075	6982/76	CARSTEN'S ROGGEN	DEU	Cultivar	Western Europe	PAS BG	P1	Carsten
76	OP076	6977/76	CARIBOU	CAN	Cultivar	North America	PAS BG	P1/P2	-
77	OP077	827/96	CHULPAN	RUS	Cultivar	Eastern Europe	PAS BG	P1/P2	-
78	OP078	8412/78	PASTOREO MASSAUX	ARG	Cultivar	South America	PAS BG	P1/P2	-
79	OP079	2691	SARATOVSKAJA 1	RUS	Cultivar	Eastern Europe	PAS BG	P1	-
80	OP080	5838/75	ZENIT	CSK	Cultivar	Central Europe	PAS BG	P1/P2	-
81	OP081	22959/86	KOROTKOSTEBEL'NAJA 69	RUS	Cultivar	Eastern Europe	PAS BG	P2	-
82	OP082	2368/73	LOVASZPATONAI	HUN	Cultivar	Central Europe	PAS BG	P1/P2	-
83	OP083	5677/75	NEMCINOVSKAJA 50	RUS	Cultivar	Eastern Europe	PAS BG	P1	-
84	OP084	2684/73	ODESSKAJA 1	UKR	Cultivar	Eastern Europe	PAS BG	P1	-
85	OP085	48/70	TULUNSKAJA ZELENOZERNAJA	RUS	Cultivar	Eastern Europe	PAS BG	P1/P2	-
86	OP086	46/70	VJATKA 2	RUS	Cultivar	Eastern Europe	PAS BG	P1/P2	-
87	OP087	14164	R 202 *(Whitcombe J. R.)*	IND	Cultivar	Southern Asia	PAS BG	P1	-
88	OP088	5799	KIRGIZSKAJA 1	KGZ	Cultivar	Central Asia	PAS BG	P1	-
89	OP089	38/70	KALUZHSKAJA 45	RUS	Cultivar	Eastern Europe	PAS BG	P2	-
90	OP090	1138/03	VOIMA	FIN	Cultivar	Northern Europe	PAS BG	P1/P2	-
91	CMP001	16273/81	Aile	ESP	Cultivated material	Southern Europe	PAS BG	P1/P2	-
92	CMP002	16104/81	Bedecin	ROM	Cultivated material	Eastern Europe	PAS BG	P1	-
93	CMP003	2676/73	Benjakonskaja	BLR	Cultivated material	Eastern Europe	PAS BG	P1	-
94	CMP004	1396/71	Brigada de Mirandela	PRT	Cultivated material	Southern Europe	PAS BG	P1/P2	-
95	CMP005	16276/81	Ceranja de Moreruela	ESP	Cultivated material	Southern Europe	PAS BG	P2	-
96	CMP006	1323/71	Grand Crouelle	FRA	Cultivated material	Western Europe	PAS BG	P1	-
97	CMP007	6171/75	Kortowskie; Kortowo	POL	Cultivated material	Central Europe	PAS BG	P1/P2	Petkus
98	CMP008	7186/76	Ludowe	POL	Cultivated material	Central Europe	PAS BG	P2	Petkus
99	CMP009	6164/75	Pulawskie Wczesne	POL	Cultivated material	Central Europe	PAS BG	P1	Petkus
100	CMP010	8858/79	R. Maly Italien 1950	ITA	Cultivated material	Southern Europe	PAS BG	P1	-
101	CMP011	17534/82	Shang Xian	CHN	Cultivated material	Western Asia	PAS BG	P1/P2	-
102	CMP012	49/70	Tacinskaja Golubaja	RUS	Cultivated material	Eastern Europe	PAS BG	P1	-
103	CMP013	7414/76	46 (Ward DJ 49)	BRA	Cultivated material	South America	PAS BG	P1/P2	-
104	CMP014	7128/76	Hungarian Giant	GBR	Cultivated material	Northern Europe	PAS BG	P1/P2	-
105	CMP015	1401/71	Castello Branco	PRT	Cultivated material	Southern Europe	PAS BG	P2	-
106	CMP016	6983/76	Centeno de La Estanzuela	URY	Cultivated material	South America	PAS BG	P2	-
107	CMP017	1308/71	Gulzower	DEU	Cultivated material	Western Europe	PAS BG	P1	-
108	CMP018	1032/71	Hania	GRC	Cultivated material	Balkans	PAS BG	P2	-

109	CMP019	41/70	Kazanskaja	RUS	Cultivated material	Eastern Europe	PAS BG	P1/P2	-
110	CMP020	8856/79	Pico Uruguay	URY	Cultivated material	South America	PAS BG	P2	-
111	CMP021	7248/76	Rheidol	GBR	Cultivated material	Northern Europe	PAS BG	P1/P2	-
112	CMP022	7410/76	45 (Ward DJ 45)	BRA	Cultivated material	South America	PAS BG	P1/P2	-
113	CMP023	1281/71	Visa	FIN	Cultivated material	Northern Europe	PAS BG	P1/P2	-
114	CMP024	7466/76	Sample B	BRA	Cultivated material	South America	PAS BG	P1/P2	-
115	CMP025	5015/75	Tiroler	AUT	Cultivated material	Western Europe	PAS BG	P1	-
116	CMP026	7344/76	Uniwersalne	POL	Cultivated material	Central Europe	PAS BG	P1	Petkus
117	CMP027	7408/76	42 (Ward DJ 42)	BRA	Cultivated material	South America	PAS BG	P1/P2	-
118	CMP028	7412/76	47 (Ward DJ 47)	BRA	Cultivated material	South America	PAS BG	P1/P2	-
119	CMP029	1317/71	Varne	SWE	Cultivated material	Northern Europe	PAS BG	P1	-
120	CMP030	5840/75	Zhitkinskaja Mestnaja	RUS	Cultivated material	Eastern Europe	PAS BG	P2	-
121	CMP031	5005/75	Otterbacher	AUT	Cultivated material	Western Europe	PAS BG	P1	-
122	CMP032	7029/76	Emory	USA	Cultivated material	North America	PAS BG	P2	-
123	CMP033	1341/71	Litvinskaja Mestnaja	LTU	Cultivated material	Eastern Europe	PAS BG	P1	-
124	CMP034	14062/80	Ashill Pearl	GBR	Cultivated material	Northern Europe	PAS BG	P1	-
125	CMP035	2266/93	Korea I	KOR	Cultivated material	Western Asia	PAS BG	P1/P2	-
126	CMP036	1400/71	Barroso	PRT	Cultivated material	Southern Europe	PAS BG	P2	-
127	CMP037	1031/71	Kastoris	GRC	Cultivated material	Balkans	PAS BG	P2	-
128	CMP038	1614/92	Irlanda I	IRL	Cultivated material	Northern Europe	PAS BG	P1/P2	-
129	CMP039	2267/93	Korean	JPN	Cultivated material	Western Asia	PAS BG	P2	-
130	CMP040	7150/76	Kenya	KEN	Cultivated material	Eastern Africa	PAS BG	P1/P2	-
131	CMP041	100/03	44 (Ward DJ 44)	BRA	Cultivated material	South America	PAS BG	P2	-
132	CMP042	1096/03	PI 237927	BRA	Cultivated material	South America	PAS BG	P2	-
133	CMP043	1098/03	38 (Ward DJ 38)	BRA	Cultivated material	South America	PAS BG	P1	-
134	CMP044	1101/03	48 (Ward DJ 48)	BRA	Cultivated material	South America	PAS BG	P1/P2	-
135	CMP045	1415/71	Miranda do Corvo	PRT	Cultivated material	Southern Europe	PAS BG	P1	-
136	CMP046	7018/76	Edelhofer Neu	AUT	Cultivated material	Western Europe	PAS BG	P1/P2	Petkus
137	LRC001	2959/76	Abruzzi	ITA	Landrace	Southern Europe	PAS BG	P1/P2	-
138	LRC002	14158/80	26b (Altefogt R.F.2)	IRN	Landrace	Southern Asia	PAS BG	P2	-
139	LRC003	14159/80	43b (Altevogt R.F)	IRN	Landrace	Southern Asia	PAS BG	P2	-
140	LRC004	14160/80	91c (Altevogt R.F.)	IRN	Landrace	Southern Asia	PAS BG	P1/P2	-
141	LRC005	14161/80	92b (Altefogt R.F.9)	IRN	Landrace	Southern Asia	PAS BG	P1/P2	-
142	LRC006	7055/76	15320 (Gentry H.S.)	IRN	Landrace	Southern Asia	PAS BG	P2	-
143	LRC007	7269/76	Jowder (Smith E.E.1040)	AFG	Landrace	Eastern Asia	PAS BG	P2	-
144	LRC008	7259/76	Jawad (Smith E.E. 312)	AFG	Landrace	Eastern Asia	PAS BG	P2	-
145	LRC009	7260/76	332 (Smith E.E.)	AFG	Landrace	Eastern Asia	PAS BG	P2	-
146	LRC010	8872	TURKOVSKAJA MESTNAJA	UKR	Landrace	Eastern Europe	PAS BG	P2	-
147	LRC011	7062/76	132 (Gray J.D.)	AFG	Landrace	Eastern Asia	PAS BG	P2	-
148	LRC012	7054/76	15188 (Gentry H.S.)	IRN	Landrace	Southern Asia	PAS BG	P1/P2	-
149	LRC013	7071/76	221 (Harlan J.R.)	AFG	Landrace	Eastern Asia	PAS BG	P1/P2	-
150	LRC014	7155/76	K1526 (Knowles 1526)	IRN	Landrace	Southern Asia	PAS BG	P2	-
151	LRC015	7159/76	K1692 (Knowles PF 1692)	IRN	Landrace	Southern Asia	PAS BG	P2	-
152	LRC016	7442/76	78	HRV	Landrace	Balkans	PAS BG	P1	-
153	LRC017	7445/76	82	YUG	Landrace	Balkans	PAS BG	P2	-
154	LRC018	7449/76	87	YUG	Landrace	Balkans	PAS BG	P1/P2	-
155	LRC019	7450/76	88	YUG	Landrace	Balkans	PAS BG	P1	-
156	LRC020	7070/76	153 (Harlan J.R)	IRN	Landrace	Southern Asia	PAS BG	P1/P2	-
157	LRC021	7443/76	80	YUG	Landrace	Balkans	PAS BG	P1	-
158	LRC022	7448/76	86	YUG	Landrace	Balkans	PAS BG	P1	-
159	LRC023	7264/76	Jaudar (Smith E.E. 743)	AFG	Landrace	Eastern Asia	PAS BG	P1	-
160	LRC024	14162/80	94b (Altefogt R.F.9)	IRN	Landrace	Southern Asia	PAS BG	P2	-
161	LRC025	7064/76	231 (Gray J.D.)	AFG	Landrace	Eastern Asia	PAS BG	P2	-
162	LRC026	7157/76	K1563 (Knowles PF 15630)	IRN	Landrace	Southern Asia	PAS BG	P1/P2	-
163	LRC027	7266/76	Kaljow (Smith E.E. 907)	AFG	Landrace	Eastern Asia	PAS BG	P1/P2	-

164	LRC028	7270/76	974 (Smith E.E.)	AFG	Landrace	Eastern Asia	PAS BG	P1/P2	-
165	LRC029	1136/03	R192	IND	Landrace	Southern Asia	PAS BG	P2	-
166	LRC030	2317/73	Muchlenviertle	AUT	Landrace	Western Europe	PAS BG	P1	-
167	LRC031	7178/76	Landsorte Tz Frumos	DEU	Landrace	Western Europe	PAS BG	P1/P2	-
168	LRC032	5803/75	Kuckucks Riesengebirgs Landroggen	DEU	Landrace	Western Europe	PAS BG	P1/P2	-
169	LRC033	7417/76	1/72	YUG	Landrace	Balkans	PAS BG	P2	-
170	LRC034	7418/76	2/72	YUG	Landrace	Balkans	PAS BG	P2	-
171	LRC035	7421/76	5/72	YUG	Landrace	Balkans	PAS BG	P1/P2	-
172	LRC036	7425/76	12/72	YUG	Landrace	Balkans	PAS BG	P1/P2	-
173	LRC037	1803/92	53c (Altevogt R.F.)	IRN	Landrace	Southern Asia	PAS BG	P2	-
174	LRC038	2209/93	1/71-4	YUG	Landrace	Balkans	PAS BG	P2	-
175	LRC039	1681/92	17040	GRC	Landrace	Balkans	PAS BG	P1/P2	-
176	LRC040	1749/92	4-I/4	BIH	Landrace	Balkans	PAS BG	P2	-
177	LRC041	1752/92	21-II/21	BIH	Landrace	Balkans	PAS BG	P1/P2	-
178	LRC042	7419/76	3/72	YUG	Landrace	Balkans	PAS BG	P1	-
179	LRC043	5008/75	Roemers Erzgebirgsroggen	DEU	Landrace	Western Europe	PAS BG	P1/P2	-
180	LRC044	8844/79	Mestnaja	YUG	Landrace	Balkans	PAS BG	P1/P2	-
181	LRC045	1806/92	128	CHL	Landrace	South America	PAS BG	P1	-
182	LRC046	1535/91	86PK1305-002	PAK	Landrace	Southern Asia	PAS BG	P2	-
183	LRC047	7063/76	206 (Gray J.D.)	AFG	Landrace	Eastern Asia	PAS BG	P1/P2	-
184	LRC048	7073/76	358 (HarlAN J.D.)	AFG	Landrace	Eastern Asia	PAS BG	P2	-
185	LRC049	7068/76	94 (Harlan J.R.)	IRN	Landrace	Southern Asia	PAS BG	P2	-
186	LRC050	7072/76	225 (Harlan J.R.)	AFG	Landrace	Eastern Asia	PAS BG	P2	-
187	LRC051	18708/83	Kaltenberger	AUT	Landrace	Western Europe	PAS BG	P2	-
188	LRC052	18709/83	Lungauer Tauern	AUT	Landrace	Western Europe	PAS BG	P2	-
189	LRC053	1780/94	ZD-2	ISR	Landrace	Western Asia	PAS BG	P2	-
190	LRC054	1781/94	ZD-3	ISR	Landrace	Western Asia	PAS BG	P2	-
191	LRC055	1751/92	19-II/19	YUG	Landrace	Balkans	PAS BG	P2	-
192	LRC056	2252/93	98b (Altevogt R.F.9)	IRN	Landrace	Southern Asia	PAS BG	P2	-
193	LRC057	2260/93	542 (Gray J.D.)	AFG	Landrace	Eastern Asia	PAS BG	P2	-
194	LRC058	2261/93	436 (Gray J.D.)	AFG	Landrace	Eastern Asia	PAS BG	P2	-
195	LRC059	1773/92	16/71-24	YUG	Landrace	Balkans	PAS BG	P2	-
196	LRC060	1544/91	86PK1262-003	PAK	Landrace	Southern Asia	PAS BG	P2	-
197	LRC061	1534/91	86PK1271-002	PAK	Landrace	Southern Asia	PAS BG	P1/P2	-
198	LRC062	1757/92	60	YUG	Landrace	Balkans	PAS BG	P1/P2	-
199	LRC063	1105/03	3115 (Gentry H.S.)	GRC	Landrace	Balkans	PAS BG	P1	-
200	LRC064	1114/03	889 (Harlan J.R.)	PAK	Landrace	Southern Asia	PAS BG	P1/P2	-
201	LRC065	1092/03	75	YUG	Landrace	Balkans	PAS BG	P1/P2	-
202	LRC066	1089/03	15/71-23	YUG	Landrace	Balkans	PAS BG	P1	-
203	LRC067	1090/03	19/72	YUG	Landrace	Balkans	PAS BG	P1	-
204	LRC068	7355/76	V/10	MKD	Landrace	Balkans	PAS BG	P1/P2	-
205	LRC069	14093/80	V/109	MKD	Landrace	Balkans	PAS BG	P1/P2	-
206	LRC070	16267/81	116/1977	PRT	Landrace	Southern Europe	PAS BG	P2	-
207	LRC071	16270/81	130/1977	PRT	Landrace	Southern Europe	PAS BG	P2	-
208	LRC072	17539/82	77 A-17	PRT	Landrace	Southern Europe	PAS BG	P1/P2	-
209	LRC073	17559/82	77 A-58	PRT	Landrace	Southern Europe	PAS BG	P2	-
210	LRC074	17684/82	78 A-674	PRT	Landrace	Southern Europe	PAS BG	P2	-
211	LRC075	17703/82	78 A-881	PRT	Landrace	Southern Europe	PAS BG	P1/P2	-
212	LRC076	2071/93	M1-72-73-175	TUR	Landrace	Western Asia	PAS BG	P2	-
213	LRC077	2074/93	M1-72-73-178	TUR	Landrace	Western Asia	PAS BG	P2	-
214	LRC078	2180/93	M1-72-73-321	TUR	Landrace	Western Asia	PAS BG	P2	-
215	LRC079	2183/93	M1-72-73-399	TUR	Landrace	Western Asia	PAS BG	P2	-
216	LRC080	7357/76	V/127	MKD	Landrace	Balkans	PAS BG	P1/P2	-
217	LRC081	14088/80	V/104	MKD	Landrace	Balkans	PAS BG	P2	-
218	LRC082	1288/97	M1-72-73-262	TUR	Landrace	Western Asia	PAS BG	P2	-

219	LRC083	1289/97	M1-72-73-263	TUR	Landrace	Western Asia	PAS BG	P2	-
220	LRC084	1636/92	M1-72-73-290	TUR	Landrace	Western Asia	PAS BG	P2	-
221	LRC085	1314/97	M1-72-73-405	TUR	Landrace	Western Asia	PAS BG	P2	-
222	LRC086	1646/92	M1-72-73-410	TUR	Landrace	Western Asia	PAS BG	P2	-
223	LRC087	16254/81	34/1977	PRT	Landrace	Southern Europe	PAS BG	P2	-
224	LRC088	16231/81	583/1978	PRT	Landrace	Southern Europe	PAS BG	P2	-
225	LRC089	16269/81	125/1977	PRT	Landrace	Southern Europe	PAS BG	P1/P2	-
226	LRC090	16271/81	136/1977	PRT	Landrace	Southern Europe	PAS BG	P2	-
227	LRC091	16237/81	611/1978	PRT	Landrace	Southern Europe	PAS BG	P2	-
228	LRC092	17556/82	77 A-49	PRT	Landrace	Southern Europe	PAS BG	P2	-
229	LRC093	17603/82	78 A-554	PRT	Landrace	Southern Europe	PAS BG	P2	-
230	LRC094	16263/81	98/1977	PRT	Landrace	Southern Europe	PAS BG	P2	-
231	LRC095	14090/80	V/106	MKD	Landrace	Balkans	PAS BG	P2	-
232	LRC096	14106/80	V/122	MKD	Landrace	Balkans	PAS BG	P2	-
233	LRC097	1119/98	M1-72-73-424	TUR	Landrace	Western Asia	PAS BG	P2	-
234	LRC098	7356/76	V/11	MKD	Landrace	Balkans	PAS BG	P1/P2	-
235	LRC099	14098/80	V/114	MKD	Landrace	Balkans	PAS BG	P2	-
236	LRC100	14102/80	V/118	MKD	Landrace	Balkans	PAS BG	P2	-
237	LRC101	1099/98	M1-72-73-375	TUR	Landrace	Western Asia	PAS BG	P2	-
238	LRC102	17555/82	77 A-48	PRT	Landrace	Southern Europe	PAS BG	P2	-
239	LRC103	17568/82	77 A-81	PRT	Landrace	Southern Europe	PAS BG	P2	-
240	LRC104	14111/80	V/127	MKD	Landrace	Balkans	PAS BG	P2	-
241	LRC105	14130/80	V/147	MKD	Landrace	Balkans	PAS BG	P2	-
242	LRC106	16230/81	576/1978	PRT	Landrace	Southern Europe	PAS BG	P2	-
243	LRC107	16243/81	642/1978	PRT	Landrace	Southern Europe	PAS BG	P2	-
244	LRC108	17576/82	77 A-100	PRT	Landrace	Southern Europe	PAS BG	P2	-
245	LRC109	17595/82	77 A-128	PRT	Landrace	Southern Europe	PAS BG	P2	-
246	LRC110	17678/82	78 A-665	PRT	Landrace	Southern Europe	PAS BG	P2	-
247	LRC111	2082/93	M1-72-73-191	TUR	Landrace	Western Asia	PAS BG	P2	-
248	LRC112	2093/93	M1-72-73-206	TUR	Landrace	Western Asia	PAS BG	P2	-
249	LRC113	2114/93	M1-72-73-238	TUR	Landrace	Western Asia	PAS BG	P2	-
250	LRC114	2121/93	M1-72-73-247	TUR	Landrace	Western Asia	PAS BG	P2	-
251	LRC115	2138/93	M1-72-73-264	TUR	Landrace	Western Asia	PAS BG	P2	-
252	LRC116	2143/93	M1-72-73-295	TUR	Landrace	Western Asia	PAS BG	P2	-
253	LRC117	17567/82	77 A-80	PRT	Landrace	Southern Europe	PAS BG	P2	-
254	LRC118	17588/82	77 A-118	PRT	Landrace	Southern Europe	PAS BG	P2	-
255	LRC119	17606/82	78 A-558	PRT	Landrace	Southern Europe	PAS BG	P2	-
256	LRC120	17654/82	78 A-627	PRT	Landrace	Southern Europe	PAS BG	P2	-
257	LRC121	14095/80	V/111	MKD	Landrace	Balkans	PAS BG	P2	-
258	LRC122	14122/80	V/138	MKD	Landrace	Balkans	PAS BG	P2	-
259	LRC123	1252/97	M1-72-73-176	TUR	Landrace	Western Asia	PAS BG	P2	-
260	LRC124	1268/97	M1-72-73-212	TUR	Landrace	Western Asia	PAS BG	P2	-
261	LRC125	1272/97	M1-72-73-228	TUR	Landrace	Western Asia	PAS BG	P2	-
262	LRC126	1071/98	M1-72-73-274	TUR	Landrace	Western Asia	PAS BG	P2	-
263	LRC127	1291/97	M1-72-73-289	TUR	Landrace	Western Asia	PAS BG	P2	-
264	LRC128	1299/97	M1-72-73-314	TUR	Landrace	Western Asia	PAS BG	P2	-
265	LRC129	1082/98	M1-72-73-333	TUR	Landrace	Western Asia	PAS BG	P2	-
266	LRC130	1643/92	M1-72-73-393	TUR	Landrace	Western Asia	PAS BG	P2	-
267	LRC131	1260/97	M1-72-73-194	TUR	Landrace	Western Asia	PAS BG	P2	-
268	LRC132	1641/92	M1-72-73-368	TUR	Landrace	Western Asia	PAS BG	P2	-
269	LRC133	17543/82	77 A-26	PRT	Landrace	Southern Europe	PAS BG	P2	-
270	LRC134	17563/82	77 A-70	PRT	Landrace	Southern Europe	PAS BG	P2	-
271	LRC135	17593/82	77 A-124	PRT	Landrace	Southern Europe	PAS BG	P2	-
272	LRC136	17655/82	78 A-628	PRT	Landrace	Southern Europe	PAS BG	P1/P2	-
273	LRC137	17675/82	78 A-662	PRT	Landrace	Southern Europe	PAS BG	P1/P2	-

274	LRC138	14107/80	V/123	MKD	Landrace	Balkans	PAS BG	P1	-
275	LRC139	14126/80	V/142	MKD	Landrace	Balkans	PAS BG	P1/P2	-
276	LRC140	17569/82	77 A-83	PRT	Landrace	Southern Europe	PAS BG	P1	-
277	LRC141	17686/82	78 A-678	PRT	Landrace	Southern Europe	PAS BG	P1/P2	-
278	LRC142	16257/81	55/1977	PRT	Landrace	Southern Europe	PAS BG	P1/P2	-
279	LRC143	17600/82	77 A-143	PRT	Landrace	Southern Europe	PAS BG	P1	-
280	LRC144	17546/82	77 A-31	PRT	Landrace	Southern Europe	PAS BG	P1	-
281	LRC145	1092/98	M1-72-73-362	TUR	Landrace	Western Asia	PAS BG	P2	-
282	LRC146	1110/98	M1-72-73-394	TUR	Landrace	Western Asia	PAS BG	P1/P2	-
283	LRC147	1114/98	M1-72-73-416	TUR	Landrace	Western Asia	PAS BG	P2	-
284	LRC148	1078/98	M1-72-73-291	TUR	Landrace	Western Asia	PAS BG	P2	-
285	LRC149	678/01	M1-72-73-203	TUR	Landrace	Western Asia	PAS BG	P2	-
286	LRC150	689/01	M1-72-73-271	TUR	Landrace	Western Asia	PAS BG	P2	-
287	LRC151	696/01	M1-72-73-307	TUR	Landrace	Western Asia	PAS BG	P2	-
288	LRC152	714/01	M1-72-73-419	TUR	Landrace	Western Asia	PAS BG	P1/P2	-
289	LRC153	2233/93	V/127	MKD	Landrace	Balkans	PAS BG	P1/P2	-
290	LRC154	17662/82	78 A-637	PRT	Landrace	Southern Europe	PAS BG	P1	-
291	LRC155	17562/82	77 A-69	PRT	Landrace	Southern Europe	PAS BG	P1/P2	-
292	OH001	na	G5959 *S. cereale* wild population from San Bernardino CA USA	USA	naturalized population	North America	Collection of A. Lukaszewski	P2	-
293	OH002	na	*S. sereal* e Snoopy E3	MEX	selection from Snoopy	North America	Collection of A. Lukaszewski	P2	-
294	OH003	na	*S. sereale* cv. Tetra Prima	USA	Cultivar	North America	Collection of A. Lukaszewski	P1/P2	Petkus
295	OH004	na	*S. sereale* cv. Musketeer	CAN	Cultivar	North America	Collection of A. Lukaszewski	P1	Petkus
296	OH005	na	*S. sereale* cv. Prima	CAN	Cultivar	North America	Collection of A. Lukaszewski	P1/P2	Petkus
297	OH006	na	*S. sereale* cv. Tetra Gator	USA	Cultivar	North America	Collection of A. Lukaszewski	P1/P2	-
298	OH007	na	*S. sereale* cv. Tetra Tenn	USA	Cultivar	North America	Collection of A. Lukaszewski	P1/P2	-
299	OH008	na	*S. sereale* cv. Imperial	USA	Cultivar	North America	Collection of A. Lukaszewski	P1/P2	Petkus
300	OH009	na	*S. sereale* cv. King II	SWE	Cultivar	Northern Europe	Collection of A. Lukaszewski	P1/P2	Petkus
301	OH010	na	*S. sereale* cv. Blanco	BRA	Cultivar	South America	Collection of A. Lukaszewski	P1/P2	-
302	OH011	na	*S. sereale* Mtzg	USA	Cultivar	North America	Collection of A. Lukaszewski	P1/P2	-
303	OB001	na	SARATOVSKAYA 7	BLR	Cultivar	Eastern Europe	SPCAF	P1	-
304	OB002	na	MARUSENYKA	BLR	Cultivar	Eastern Europe	SPCAF	P1	-
305	OB003	na	IVAN	BLR	Cultivar	Eastern Europe	SPCAF	P1	-
306	OD001	na	DAŃKOWSKIE ZŁOTE	POL	Cultivar	Central Europe	Danko	P1/P2	Petkus
307	OD002	na	DAŃKOWSKIE ZŁOTE/73	POL	Cultivar	Central Europe	Danko	P1/P2	Petkus
308	OD005	na	DAŃKOWSKIE NOWE	POL	Cultivar	Central Europe	Danko	P1	Petkus
309	OD006	na	KIER	POL	Cultivar	Central Europe	Danko	P1	Petkus
310	OD007	na	WALET	POL	Cultivar	Central Europe	Danko	P1	Petkus
311	OD008	na	MOTTO	POL	Cultivar	Central Europe	Danko	P1	Petkus
312	OD009	na	SKAT	POL	Cultivar	Central Europe	Danko	P1	Petkus
313	OD010	na	DAŃKOWSKIE AMBER	POL	Cultivar	Central Europe	Danko	P1	Petkus
314	OD011	na	WARKO	POL	Cultivar	Central Europe	Danko	P1	Petkus
315	OD012	na	AMILO	POL	Cultivar	Central Europe	Danko	P1	Petkus
316	OD013	na	DAŃKOWSKIE DIAMENT	POL	Cultivar	Central Europe	Danko	P1	Petkus
317	OD014	na	DL 6 R	POL	Cultivar	Central Europe	Danko	P1	Petkus
318	OF001	na	Riihi	FIN	Cultivar	Northern Europe	Boreal	P1	-
319	OF002	na	Reetta	FIN	Cultivar	Northern Europe	Boreal	P1	-
320	OL001	na	BALISTIC F1	POL	Cultivar	Central Europe	KWS Lochow	P1	-
321	OL002	na	BELLAMI F1	POL	Cultivar	Central Europe	KWS Lochow	P1	-
322	OL003	na	BRASETTO F1	POL	Cultivar	Central Europe	KWS Lochow	P1	-
323	OL004	na	GONELLO F1	POL	Cultivar	Central Europe	KWS Lochow	P2	-
324	OL005	na	PALAZZO F1	POL	Cultivar	Central Europe	KWS Lochow	P1	-
325	OL006	na	VISELLO F1	POL	Cultivar	Central Europe	KWS Lochow	P1	-
326	OL008	na	GUTTINO F1	DEU	Cultivar	Western Europe	KWS Lochow	P1	-
327	OL009	na	KWS MAGNIFICO F1	DEU	Cultivar	Western Europe	KWS Lochow	P1	-
328	OL010	na	RECRUT F1	DEU	Cultivar	Western Europe	KWS Lochow	P1	-

329	OL011	na	SELLINO F1	DEU	Cultivar	Western Europe	KWS Lochow	P1	-
330	OL012	na	WIBRO	POL	Cultivar	Central Europe	IHAR Smolice	P1	Petkus
331	OL013	na	HEGRO	POL	Cultivar	Central Europe	IHAR Smolice	P1	Petkus
332	OL014	na	STACH F1	POL	Cultivar	Central Europe	IHAR Smolice	P1	-
333	OL015	na	KONTO	POL	Cultivar	Central Europe	IHAR Smolice	P1	Petkus
334	OL016	na	Agrikolo	POL	Cultivar	Central Europe	IHAR Smolice	P1	Petkus
335	OL017	na	Bosmo	POL	Cultivar	Central Europe	IHAR Smolice	P1	Petkus
336	OL018	na	Słowiańskie	POL	Cultivar	Central Europe	IHAR Smolice	P1	Petkus
337	OL019	na	Rostockie	POL	Cultivar	Central Europe	IHAR Smolice	P1	Petkus
338	OL020	na	Bojko (jare)	POL	Cultivar	Central Europe	IHAR Smolice	P1	-
339	PHR001	na	GRADAN F1	POL	Cultivar	Central Europe	PHR	P1	-
340	PHR002	na	ARANT	POL	Cultivar	Central Europe	PHR	P1	Petkus
341	PHR003	na	PASTAR	POL	Cultivar	Central Europe	PHR	P1	-
342	SZK.101	na	Szk101	POL	Breeding strain	Central Europe	Danko	P1	Petkus
343	SZK.35	na	Szk35	POL	Breeding strain	Central Europe	Danko	P1	Petkus
344	SZK.36	na	Szk36	POL	Breeding strain	Central Europe	Danko	P1	Petkus
345	SZK.41	na	Szk41	POL	Breeding strain	Central Europe	Danko	P1	Petkus
346	SZK.42	na	Szk42	POL	Breeding strain	Central Europe	Danko	P1	Petkus
347	SZK.43	na	Szk43	POL	Breeding strain	Central Europe	Danko	P1	Petkus
348	SZK.44	na	Szk44	POL	Breeding strain	Central Europe	Danko	P1	Petkus
349	SZK.45	na	Szk45	POL	Breeding strain	Central Europe	Danko	P1	Petkus
350	SZK.46	na	Szk46	POL	Breeding strain	Central Europe	Danko	P1	Petkus
351	SZK.51	na	Szk51	POL	Breeding strain	Central Europe	Danko	P1	Petkus
352	SZK.54	na	Szk54	POL	Breeding strain	Central Europe	Danko	P1	Petkus
353	SZK.61	na	Szk61	POL	Breeding strain	Central Europe	Danko	P1	Petkus
354	SZK.62	na	Szk62	POL	Breeding strain	Central Europe	Danko	P1	Petkus
355	SZK.73	na	Szk73	POL	Breeding strain	Central Europe	Danko	P1	Petkus
356	SZK.74	na	Szk74	POL	Breeding strain	Central Europe	Danko	P1	Petkus
357	SZK.75	na	Szk75	POL	Breeding strain	Central Europe	Danko	P1	Petkus
358	SZK.81	na	Szk81	POL	Breeding strain	Central Europe	Danko	P1	Petkus
359	SZK.82	na	Szk82	POL	Breeding strain	Central Europe	Danko	P1	Petkus
360	SZK.83	na	Szk83	POL	Breeding strain	Central Europe	Danko	P1	Petkus
361	SZK.84	na	Szk84	POL	Breeding strain	Central Europe	Danko	P1	Petkus
362	SZK.85	na	Szk85	POL	Breeding strain	Central Europe	Danko	P1	Petkus
363	SZK.86	na	Szk86	POL	Breeding strain	Central Europe	Danko	P1	Petkus
364	SZK.87	na	Szk87	POL	Breeding strain	Central Europe	Danko	P1	Petkus
365	SZK.89	na	Szk89	POL	Breeding strain	Central Europe	Danko	P1	Petkus
366	SZK.91	na	Szk91	POL	Breeding strain	Central Europe	Danko	P1	Petkus
367	SZK.92	na	Szk92	POL	Breeding strain	Central Europe	Danko	P1	Petkus

PAS BG: Polish Academy of Sciences Botanical Garden-Center for Biological Diversity Conservation in Powsin
Boreal: Boreal Plant Breeding Ltd
Danko: Danko Plant Breeders Ltd
IHAR Smolice: Hodowla Roślin Smolice Sp. z o. o. Grupa IHAR
PHR: Poznańska Hodowla Roślin -Poznań Plant Breeders Ltd.
SPCAF: Scientific and Practical Centre of Bellorussian NAS for Arable Farming
