# Supplementary material for: Assessment of Genetic Diversity in Secale cereale Based on SSR Markers
Source: Plant Mol Biol Report. 2015 Jun 6;34:37–51. doi: 10.1007/s11105-015-0896-4 (PMC4722074; doi:10.1007/s11105-015-0896-4)
Supplement: Supplementary file 2 — SSR markers used in preliminary analyses. (PDF 83 kb) [file 11105_2015_896_MOESM2_ESM.pdf]

No.	SSR marker	Chromosome	SSR motif	Forward primer	Reverse primer	max. product size (bp)	min. product size (bp)	Reference
1	SCM002	6R	(GT)10	GATGACTATGACTACCAGGATGAA	GGAGTGAGAAGGCCGAGAAG	118	107	Saal and Wricke, 1999
2	SCM005	3R	(GA)16	TCGCGATACATCAAGATCGTG	CTAGCATCGACGTAACCCTTT	228	-	Saal and Wricke, 1999
3	SCM009	1R	(GT)8	TGACAACCCCCTTTCCCTCGT	TCATCGACGCTAAGGAGGACCC	255	205	Saal and Wricke, 1999
4	SCM021	1R	(AAC)5	TGCCAAGTCCTCACAGTGTT	GGTCGGGAACTTTGATCTCTT	375	340	Hackauf and Wehling 2002
5	SCM028	6R	(GT)26	CTGGTCCTGGTCTGGTGGGTC	CGCATCGGGTGTGTCGCATAC	130	128	Saal and Wricke, 1999
6	SCM029	5R	(ACC)5	CGTCTCGTCGCGTAAAAACTG	AGATCTGGGGCGACTGA	110	-	Hackauf and Wehling 2002
7	SCM039	1R	(GT)8(GC)6…(GT)53	GACCTCAGTGGAGCCTCTAGGT	GGACATCTGCCGTGACAATACC	260	240	Saal and Wricke, 1999
8	SCM040	7R	(GT)18	CCCTTCAGCGGTCATTGTTG	CACATCTTGGGCCTGACACC	98	-	Saal and Wricke, 1999
9	SCM041	2R	(AGC)5…(AAGAG)5	TGATAGCGGGGGGAAGAG	GCTGCTTGTGCTTGAAGAGAA	160	131	Hackauf and Wehling 2002
10	SCM043	2R	(GT)11	CTAGGGGATTACAGGGAGGGCA	GTTCCCTTGTCCTACTCGTTACCG	102	-	Saal and Wricke, 1999
11	SCM047	4R	(AGG)6	CCTATGGACGCGACATCACTG	CGCACGTGGTTCACAAGCA	176	163	Hackauf and Wehling 2002
12	SCM049	7R	(AGG)6	TGGGTTCGATTTGTTGCTGTT	CGAGACGAGGCAGGATTT	500	495	Hackauf and Wehling 2002
13	SCM050	7R	(AGG)5	TCGGAGGCAGCGACCACCA	TGCCAGGAACCAGGTTCTCTG	145	98	Hackauf and Wehling 2002
14	SCM063	7R	(CCG)5	CGACTTCGAGGGCAGGAATGA	ATCCCGGGGATGAAGTGCAG	250	224	Hackauf and Wehling 2002
15	SCM065	-	(CA)7…(CA)8	CCCGTCCTTCAGTTATGTATGG	TTTGATCGATGAAGAGAGCCA	310	267	Saal and Wricke, 1999
16	SCM069	2R	(CA)10…(CA)7	CTACCTGCTGTTCCCATTTGG	GTGTGTAGAAGATGTTGTCCTGG	193	-	Saal and Wricke, 1999
17	SCM073	2R	(CCT)7…(CCG)7	GCCAAACCCTACCCTCCT	CCGAGCGTCACGTTCTC	228	163	Hackauf and Wehling 2002
18	SCM075	2R	(CA)7(CT)15…(CA)10	TTTTCTATCTCAGCGATTCATGC	TCCTGAGATCAAGTGCGTGTG	192	158	Saal and Wricke, 1999
19	SCM085	5R	(CGG)8	TTCTCCCTAGCGGATCCACA	CCTCGCCCGCCTTCTTGA	470	280	Hackauf and Wehling 2002
20	SCM086	7R	(GT)20	CAGATAGATGGGTGTTGTGCG	CTCTTCTCGACATCCACACTCC	110	-	Saal and Wricke, 1999
21	SCM095	3R	(GCG)7	GATCCCGCTGAATCCTCAAAC	AAACATTTTCGCCTCCAATCC	125	110	Hackauf and Wehling 2002
22	SCM101	4R	(CT)18	GCCAGCCGCCACCTTAATTG	AGCCCAACTCTTTCGTGCATG	200	150	Saal and Wricke, 1999
23	SCM102	3R	(AG)27	AACAAGTGCAGAACTCGCGT	CAGAAAGTCTTGGGGCCAG	175	-	Saal and Wricke, 1999
24	SCM104	-	(TG)9	GATAGTGGGACATTTGGGTACG	GCCATTCCCCATTAGTACGG	199	161	Saal and Wricke, 1999
25	SCM107	1R	(GCC)5	CCCGAACCCTAACCCTAAAAC	AGCTCCTTCTCCTCCCTGAC	252	232	Hackauf and Wehling 2002
26	SCM109	5R	(GT)9	AACCCCCTTTCGTACCTTGT	TAAAGCAAACCACCAGAGCC	145	127	Saal and Wricke, 1999
27	SCM111	-	(GT)9…(GT)26	TGCAGATTCACTTATCAACACACAC	TCTGATATCTTTCCAACGGCGT	123	108	Saal and Wricke, 1999
28	SCM112	3R	(GGC)5	CCACTGCTCCTCCCAAAAG	CCCCTGCTTGTCCACATTATC	410	375	Hackauf and Wehling 2002
29	SCM118	2R	(TCT)5	CAAGCCAGCCTCTTCTTCTTC	GAGCGTGGAGATGAACTCG	166	145	Hackauf and Wehling 2002
30	SCM138	5R	(AC)23	ATAGCCGCAGATGGTTGAGGAC	GAGAAGTCTACAAATCAAGGGGGC	128	102	Saal and Wricke, 1999
31	SCM139	4R	(ATCT)3	TACCACCGCTCTCCTCGACCT	GGTGTGCTGCTCCATGTTCAG	142	126	Hackauf and Wehling 2002
32	SCM152	5R	(AG)7	CGGAGCAGCAGAGCAAGAGA	ATGTAGCCGAGGATGGTGAGC	391	320	Hackauf and Wehling 2002
33	SCM155	4R	(AAG)5	TTCTTCTTCGCTACGCACACC	TCCGGCCACTACCACATCTT	243	218	Hackauf and Wehling 2002
34	SCM159	5R	(GAAA)5	CGGGCCGGAAACACAAAA	GGCGGGAAGGAAAAACAGAAA	123	109	Hackauf and Wehling 2002
35	SCM162	3R	(CCG)7	TGGCATGGTTGGGCATTGTTC	GAGCCGGCAAAGGAGCAGAGT	197	128	Hackauf and Wehling 2002
36	SCM168	6R	(CGG)5	ACGCGTCTCTGACCATGAAGC	CAGCAACGCATCGACTGAGC	130	103	Hackauf and Wehling 2002
37	SCM171	1R	(GGC)6	TCCCGAAACACTACAGGTTGA	AGGCCTAGGACCCGAACA	223	216	Hackauf and Wehling 2002
38	SCM172	5R	(CTA)5…(GGAA)4	CCTGCATCGATCGATATATCATAA	ATGTCAAGTCATCTTCACCA	160	138	Hackauf and Wehling 2002
39	SCM177	1R	(GGT)5	AGAGCACAACAGGACCCTACA	GCGTGCACCATTTCCAC	400	360	Hackauf and Wehling 2002
40	SCM179	1R	(TAG)5	ACGCCTCAACCATCACGAGAA	TCCAACTACCCCGAGTGCT	150	130	Hackauf and Wehling 2002
41	SCM180	6R	(GT)6(GT)7	GTTTCGTCCCCGTTGCCATC	ACGTGTCGCTTTCCATTGCCC	145	138	Saal and Wricke, 1999
42	SCM304	6R	(CA)36	CATCGGATCACATTCACTTAGTTCTG	TAACGCCACCACCAAGCCTTC	250	224	Saal and Wricke, 1999
