# Supplementary figures and images for: Assessment of Genetic Diversity in Secale cereale Based on SSR Markers
Source: Plant Mol Biol Report. 2015 Jun 6;34:37–51. doi: 10.1007/s11105-015-0896-4 (PMC4722074; doi:10.1007/s11105-015-0896-4)

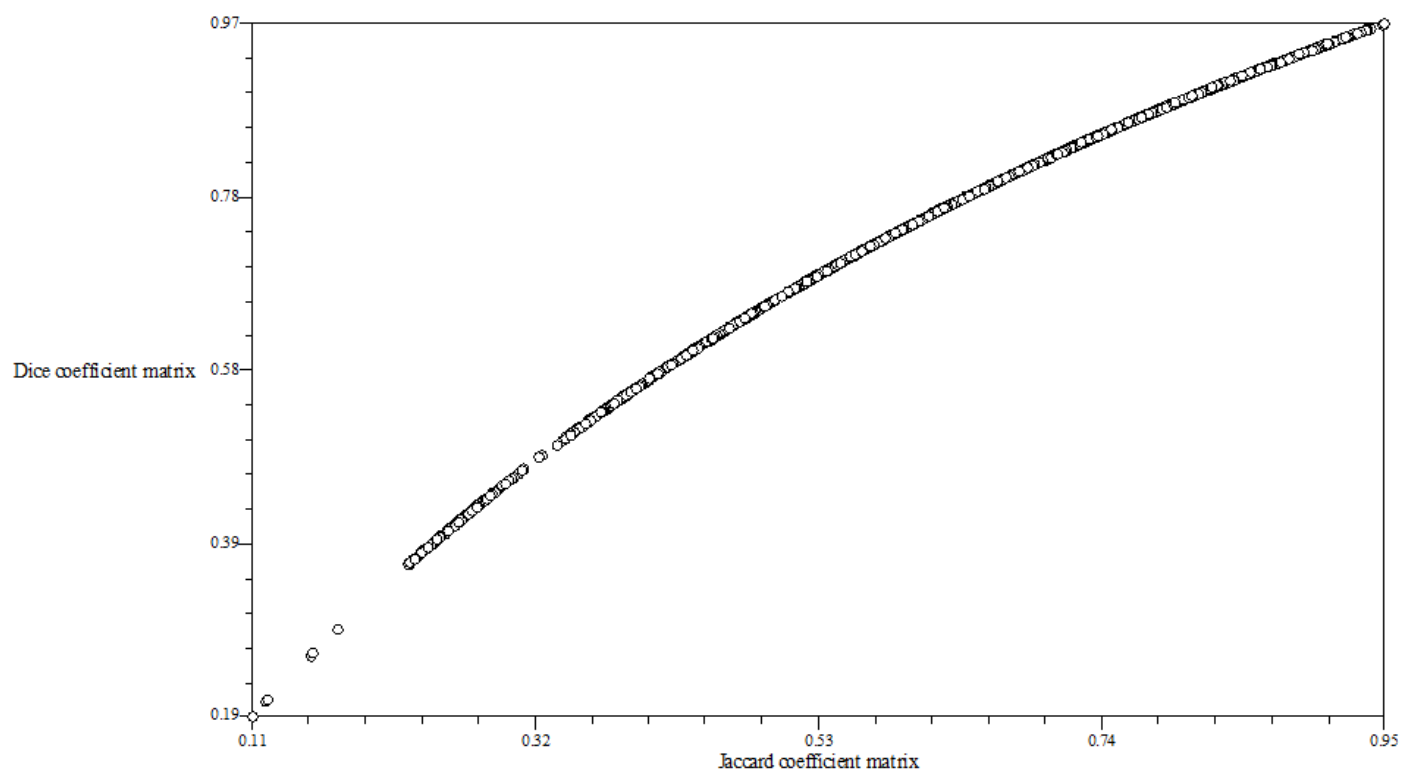

Supplement: Supplementary file 3 — The result of Mantel test showing a strong positive correlation between Jaccard and Dice coefficient. (PDF 541 kb) [file 11105_2015_896_MOESM3_ESM.pdf]

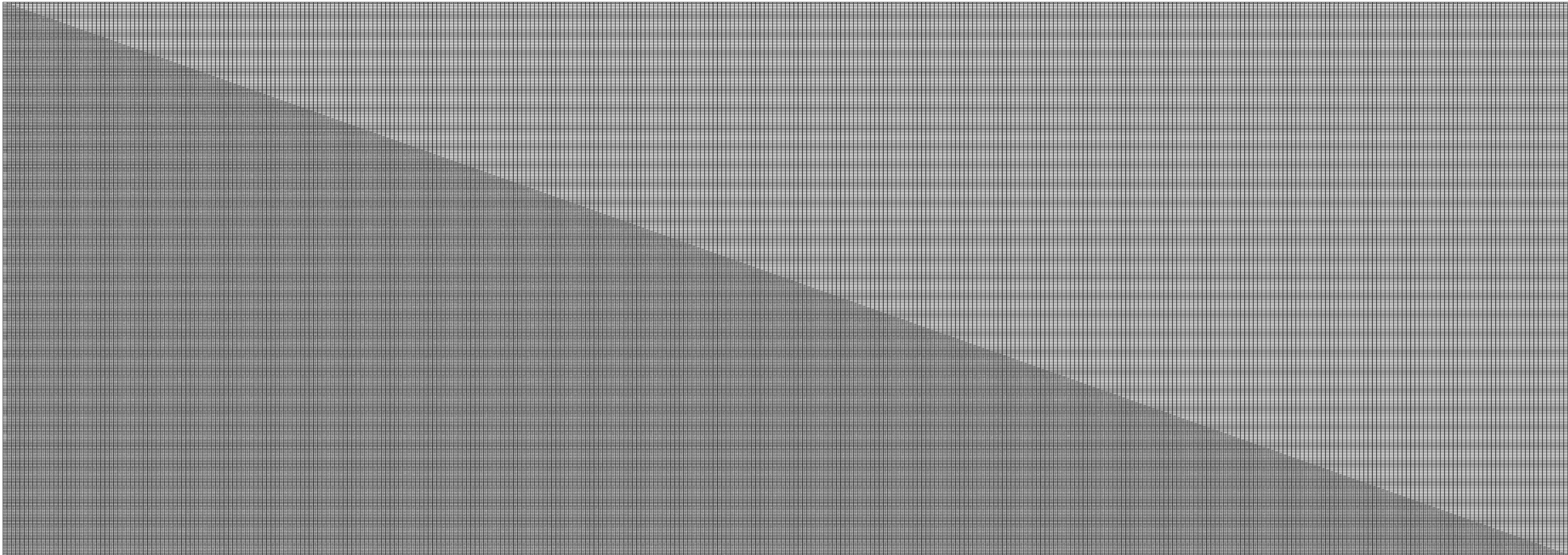

Supplement: Supplementary file 4 — GS values (according to the Jaccard coefficient) for pairs of accessions. (PDF 6710 kb) [file 11105_2015_896_MOESM4_ESM.pdf]

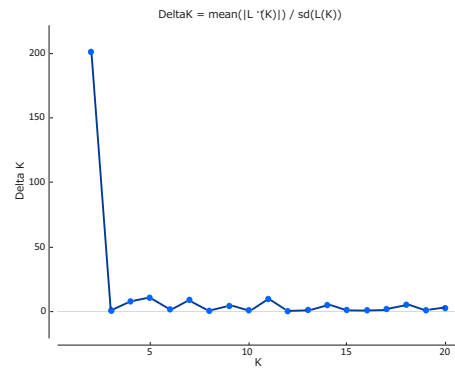

Supplement: Supplementary file 5 — Delta K values (number of populations assumed) ranging from 1 to 20. (PDF 1347 kb) [file 11105_2015_896_MOESM5_ESM.pdf]

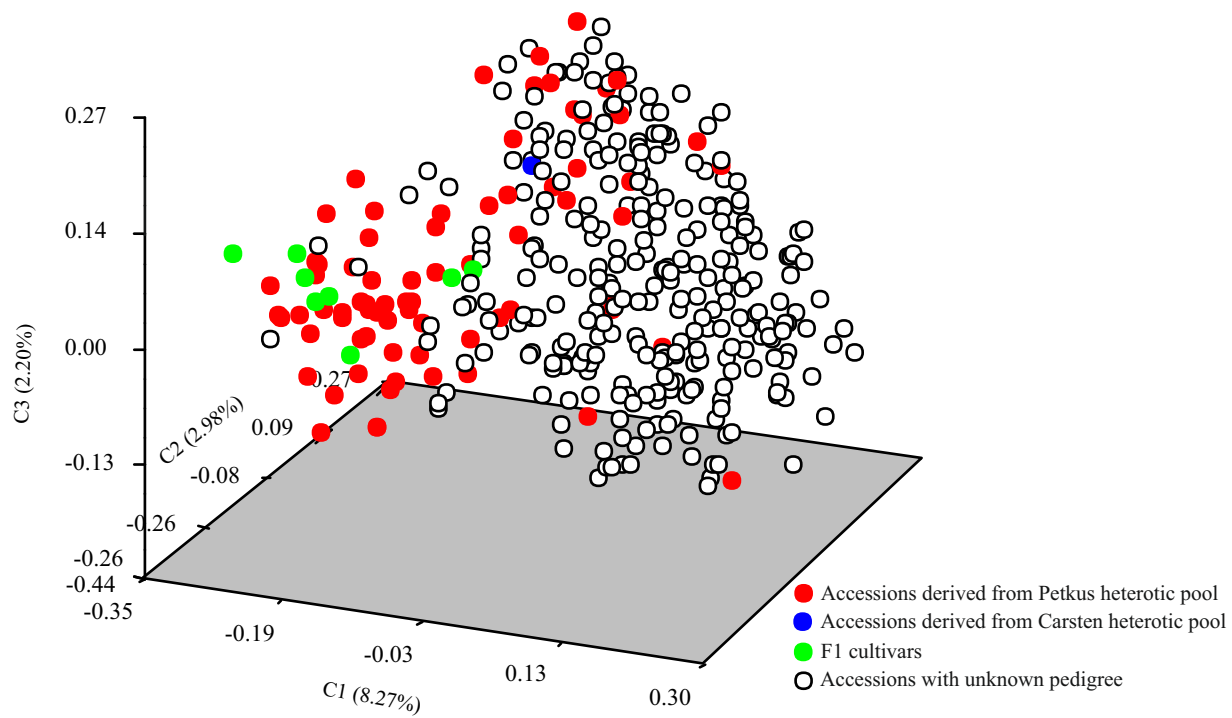

Supplement: Supplementary file 7 — PCoA of 367 rye accessions based on 22 SSR markers. Accessions labeled according to their pedigree. (PDF 1369 kb) [file 11105_2015_896_MOESM7_ESM.pdf]
