# Supplementary material for: Assessment of Genetic Diversity in Secale cereale Based on SSR Markers
Source: Plant Mol Biol Report. 2015 Jun 6;34:37–51. doi: 10.1007/s11105-015-0896-4 (PMC4722074; doi:10.1007/s11105-015-0896-4)
Supplement: Supplementary file 6 — Results of Analysis of Molecular Variance. (PDF 34 kb) [file 11105_2015_896_MOESM6_ESM.pdf]

1) Results of Analysis of Molecular Variance for two model based populations indicated by STRUCTURE

**Pop**	P1	P2
**n**	144	126
**SSWP**	1999.514	1973.675

Summary AMOVA Table

**Source**	**df**	**SS**	**MS**	**Est. Var.**	**%**
**Among Po**	1	330.052	330.052	2.345	14%
**Within Po**	268	3973.188	14.825	14.825	86%
**Total**	269	4303.241		17.171	100%

**Stat**	**Value**	**P(rand >= data)**
**PhiPT**	0.137	0.001

PhiPT = AP / (WP + AP) = AP / TOT
Key: AP = Est. Var. Among Pops, WP = Est. Var. Within Pops

2) Results of Analysis of Molecular Variance for accession grouped according to geographic origin

**Pop**	North America	South America	Eastern Asia	Southern Asia	Western Asia	Balkans	Southern Europe	Northern Europe	Eastern Europe	Western Europe	Central Europe
**n**	17	16	14	20	42	43	47	13	41	27	85
**SSWP**	282.353	229.188	174.929	260.300	602.619	581.093	699.149	162.538	556.317	435.333	1225.235

Summary AMOVA Table

**Source**	**df**	**SS**	**MS**	**Est. Var.**	**%**
**Among Po**	10	545.146	54.515	1.247	8%
**Within Po**	354	5209.054	14.715	14.715	92%
**Total**	364	5754.200		15.962	100%

**Stat**	**Value**	**P(rand >= data)**
**PhiPT**	0.078	0.001

PhiPT = AP / (WP + AP) = AP / TOT
Key: AP = Est. Var. Among Pops, WP = Est. Var. Within Pops

Pairwise Population Linearized PhiPT Values

North America	South America	Eastern Asia	Southern Asia	Western Asia	Balkans	Southern Europe	Northern Europe	Eastern Europe	Western Europe	Central Europe	
0.000											North America
0.041	0.000										South America
0.102	0.057	0.000									Eastern Asia
0.085	0.078	0.011	0.000								Southern Asia
0.103	0.088	0.087	0.074	0.000							Western Asia
0.074	0.029	0.018	0.059	0.092	0.000						Balkans
0.068	0.038	0.073	0.083	0.053	0.045	0.000					Southern Europe
0.033	0.003	0.078	0.101	0.117	0.039	0.063	0.000				Northern Europe
0.042	0.046	0.122	0.108	0.119	0.051	0.062	0.045	0.000			Eastern Europe
0.040	0.026	0.087	0.087	0.126	0.040	0.053	0.012	0.013	0.000		Western Europe
0.074	0.095	0.165	0.157	0.211	0.102	0.127	0.034	0.071	0.033	0.000	Central Europe

Linearized PhiPT Values below diagonal.

3) Results of Analysis of Molecular Variance for accession grouped according to source of seeds and improvement status

**Pop**	Cultivars PAS BG	Cultivated materials PAS BG	Landraces PAS BG	Collection of A. Lukaszewski	Cultivars from breeding compan	Breeding strains from Danko
**n**	90	46	155	11	39	26
**SSWP**	1203.878	616.391	2273.200	175.727	581.154	232.577

Summary AMOVA Table

**Source**	**df**	**SS**	**MS**	**Est. Var.**	**%**
**Among Po**	5	651.283	130.257	2.172	13%
**Within Po**	361	5082.927	14.080	14.080	87%
**Total**	366	5734.210		16.253	100%

**Stat**	**Value**	**P(rand >= data)**
**PhiPT**	0.134	0.001

PhiPT = AP / (WP + AP) = AP / TOT

Key: AP = Est. Var. Among Pops, WP = Est. Var. Within Pops

Pairwise Population PhiPT Values

Cultivars PAS BG	Cultivated materials PAS BG	Landraces PAS BG	Collection of A. Lukaszewski	Cultivars from breeding compan	Breeding strains from Danko	
0.000						Cultivars PAS BG
0.135	0.000					Cultivated materials PAS BG
0.115	0.092	0.000				Landraces PAS BG
0.272	0.305	0.204	0.000			Collection of A. Lukaszewski
0.183	0.215	0.200	0.174	0.000		Cultivars from breeding companies
0.250	0.247	0.253	0.331	0.120	0.000	Breeding strains from Danko

Phi'PT Values below diagonal.
